# Phytochemical Characterization and Integrated In Vitro Cellular Evaluation of Immunomodulatory, Cytotoxic, Genotoxic Activities and In Vivo Assessment of the Antioxidant and Anti-Arthritic Activities of *Urtica urens* L. Ethanolic Extract

**DOI:** 10.3390/plants15193018

**Published:** 2026-10-01

**Authors:** Hamza Kemchache, Hanane Khither, Khalil Kaouane, Soraya Madoui, Amina Safsaf, Khalida Hammoudi, Stefania Ponticelli, Martina Dentato, Alessia Postiglione, Daniela Rigano, Carmina Sirignano, Viviana Maresca, Chawki Bensouici

**Affiliations:** 1 Laboratory of Applied Biochemistry, Faculty of Nature and Life Sciences, Ferhat Abbas University Setif -1-, Setif 19000, Algeria; hamza.kemchache@univ-setif.dz (H.K.); khither.hanane@univ-setif.dz (H.K.); khalil.kaouane@univ-setif.dz (K.K.); madoui_soraya85@yahoo.fr (S.M.); 2 Pathology Laboratory, Setif University Hospital, Setif 19000, Algeria; aminasafsaf1984@gmail.com; 3 Faculty of Medecine, Ferhat Abbas University Setif -1-, Setif 19000, Algeria; hammoudi.k85@gmail.com; 4 Department of Biology, University of Naples Federico II, Via Cinthia 4, 80126 Napoli, Italy; ste.ponticelli@studenti.unina.it (S.P.); martina.dentato@unina.it (M.D.); alessia.postiglione@unina.it (A.P.); 5 Department of Pharmacy, School of Medicine and Surgery, University of Naples Federico II, Via Domenico Montesano 49, 80131 Naples, Italy; daniela.rigano@unina.it (D.R.); carmina.sirignano@unina.it (C.S.); 6 Department of Life Sciences, Health and Health Professions, University of Rome “Link Campus”, 00165 Rome, Italy; 7 National Center for Biotechnology Research, Ali Mendjli, Nouvelle Ville UV 03, Constantine 25011, Algeria; c.bensouici@crbt.dz

**Keywords:** *Urtica urens* L., rheumatoid arthritis, antioxidant activity, anti-arthritic activity, oxidative stress

## Abstract

Rheumatoid arthritis (RA) is a chronic autoimmune disease characterized by persistent synovial inflammation, oxidative stress, and progressive joint destruction. Given the limitations and adverse effects of current therapies, medicinal plants represent a valuable source of novel anti-inflammatory agents. This study evaluated the phytochemical composition, antioxidant, immunomodulatory, and anti-arthritic activities of an ethanolic extract of *Urtica urens* (UUEE). LC-ESI-HRMS analysis identified forty metabolites, mainly hydroxycinnamic acid derivatives, flavonoids, terpenoid glycosides, and oxidized fatty acids. Antioxidant activity was assessed using DPPH, ABTS, and CUPRAC assays together with total phenolic and flavonoid contents. Anti-arthritic activity was investigated by inhibition of heat-induced bovine serum albumin denaturation, while cytotoxicity and genotoxicity were evaluated in HeLa and HaCaT cells. Immunomodulatory activity was assessed by analyzing IL-6 and IL-1β expression in zymosan-stimulated HaCaT cells. Therapeutic efficacy was validated in a Freund’s complete adjuvant-induced arthritis model in Wistar rats. UUEE showed strong antioxidant activity, concentration-dependent inhibition of protein denaturation, low cytotoxicity up to 100 μg/mL, downregulation of pro-inflammatory cytokines, and significant in vivo anti-arthritic effects, reducing oxidative stress, histopathological damage, and arthritis severity while improving hematological and biochemical parameters. These findings support *U. urens* as a promising source of bioactive compounds for RA management.

## 1. Introduction

Rheumatoid arthritis (RA) is a chronic systemic autoimmune disease characterized by persistent synovial inflammation, progressive cartilage destruction, and irreversible bone erosion, ultimately leading to chronic pain, joint deformity, disability, and reduced quality of life [1,2,3]. Affecting approximately 0.5–1% of the global population, RA represents one of the most common inflammatory rheumatic diseases and imposes a substantial socioeconomic burden due to its chronic course and lifelong therapeutic management [2,3]. Although its precise etiology remains incompletely understood, RA arises from a complex interplay between genetic predisposition, environmental triggers, and immune dysregulation, resulting in persistent inflammation and progressive joint damage [4].

Oxidative stress is now recognized as a central mechanism in RA pathogenesis, closely linked to chronic inflammation and disease progression [4,5,6]. Activated macrophages, neutrophils, and synovial fibroblasts generate excessive reactive oxygen species (ROS), overwhelming endogenous antioxidant defenses and promoting lipid peroxidation, protein oxidation, mitochondrial dysfunction, and DNA damage [5,6]. In addition to causing direct tissue injury, ROS activate redox-sensitive signaling pathways that stimulate the production of pro-inflammatory cytokines, including tumor necrosis factor-α (TNF-α), interleukin-1β (IL-1β), and interleukin-6 (IL-6), thereby sustaining synovial hyperplasia, cartilage degradation, and bone resorption [4,5,6,7,8]. Because oxidative stress and inflammation mutually amplify each other, therapeutic approaches capable of simultaneously modulating both processes have emerged as promising strategies for the management of RA.

Current treatment relies on non-steroidal anti-inflammatory drugs (NSAIDs), corticosteroids, conventional disease-modifying anti-rheumatic drugs (DMARDs), and biological therapies [9,10]. Although these treatments have significantly improved disease management, their long-term use is frequently associated with gastrointestinal, hepatic, renal, cardiovascular, and immunological adverse effects, while a considerable proportion of patients fail to achieve sustained remission or progressively develop therapeutic resistance [11]. These limitations have renewed interest in medicinal plants as complementary therapeutic resources because they contain a wide variety of naturally occurring bioactive compounds capable of simultaneously targeting multiple molecular pathways involved in chronic inflammation and oxidative stress [11].

Among these, *Urtica urens* L. (Urticaceae), commonly known as annual stinging nettle, has long been employed in traditional medicine for the treatment of inflammatory disorders, rheumatism, arthritis, skin diseases, urinary tract infections, and gastrointestinal complaints [12,13,14]. Phytochemical studies have shown that *U. urens* is particularly rich in phenolic acids, including caffeic acid, chlorogenic acid, and 5-O-caffeoylquinic acid, as well as flavonoids such as quercetin, kaempferol, and isorhamnetin glycosides [15]. These compounds possess well-documented antioxidants, anti-inflammatory, and immunomodulatory properties and are believed to underlie many of the pharmacological activities attributed to the plant. Indeed, previous investigations have demonstrated that *U. urens* extracts exhibit remarkable antioxidant capacity and reduce inflammatory responses in experimental models of acute inflammation [16,17]. However, despite these encouraging findings, comprehensive evidence linking the phytochemical composition of *U. urens* with its anti-arthritic efficacy and underlying immunomodulatory mechanisms remains scarce.

Because IL-1β and IL-6 are key mediators of synovial inflammation and major therapeutic targets in rheumatoid arthritis, evaluating their modulation provides valuable insight into the anti-inflammatory potential of natural products. Likewise, integrating phytochemical characterization with cellular and in vivo functional studies represents an effective strategy for establishing composition–activity relationships and identifying metabolites responsible for therapeutic effects [18,19].

Besides efficacy, the safety profile of medicinal plant extracts deserves careful consideration. Although species belonging to the genus *Urtica* have long been consumed as foods and traditional herbal remedies, plant extracts are chemically complex mixtures whose biological effects are often concentration dependent. While moderate concentrations may exert antioxidant and cytoprotective effects, higher doses can induce cytotoxicity or genomic instability. Therefore, complementary evaluation of cell viability and DNA integrity is essential to define biologically active yet safe concentration ranges before considering potential therapeutic applications [20,21].

Therefore, the present study aimed to provide a comprehensive evaluation of the ethanolic extract of *U. urens* (UUEE) through an integrated phytochemical, pharmacological, toxicological, and immunomodulatory approach. The metabolite profile was characterized by liquid chromatography coupled with high-resolution electrospray ionization mass spectrometry (LC-ESI-HRMS). Antioxidant capacity was assessed using DPPH, ABTS, and CUPRAC assays together with the determination of total phenolic and flavonoid contents. The anti-arthritic activity of UUEE was initially investigated through inhibition of heat-induced bovine serum albumin denaturation and subsequently validated in a Complete freund’s adjuvant (CFA)-induced arthritis model in Wistar rats. Cytotoxicity and genotoxicity were evaluated in HeLa and HaCaT cells using the CCK-8 and alkaline comet assays, respectively, while the immunomodulatory activity of UUEE was investigated in zymosan-stimulated HaCaT keratinocytes by assessing the gene expression of the pro-inflammatory cytokines IL-6 and IL-1β through RT-qPCR. Therapeutic efficacy was further evaluated by monitoring clinical signs of arthritis, oxidative stress biomarkers, hematological and biochemical parameters, and histopathological alterations in joint and liver tissues.

To the best of our knowledge, this is the first study to comprehensively investigate the therapeutic potential of *U. urens* by integrating LC-ESI-HRMS metabolite profiling with antioxidant characterization, cytotoxicity and genotoxicity assessment, immunomodulatory analysis, in vitro anti-arthritic evaluation, and in vivo validation in a CFA-induced rheumatoid arthritis model. Collectively, these findings provide new mechanistic insights into the pharmacological properties of *U. urens* and support its potential as a promising source of bioactive compounds for the development of complementary therapeutic strategies against rheumatoid arthritis.

## 2. Results

### 2.1. LC-ESI-HRMS Profiling of UUEE

The phytochemical profile of the UUEE was investigated by LC-ESI-HRMS operating in negative ionization mode, allowing the tentative annotation of 40 metabolites belonging to different phytochemical classes, including organic acids, hydroxycinnamic acid derivatives, flavonoids, terpenoid glycosides, oxylipins, and oxidized fatty acids (Table 1). Compound annotation was based on accurate mass measurements, molecular formula prediction, MS/MS fragmentation patterns, and comparison with literature data.

Among the earliest eluting metabolites, quinic acid (**1**) was identified together with its esterified derivatives, namely, 5-*O*-caffeoylquinic acid (**3**) and *p*-coumaroylquinic acid (**5**). These compounds represent key intermediates of the phenylpropanoid pathway and are frequently reported in *Urtica* species [22]. Their characteristic product ions, including *m*/*z* 191 and 173 for the quinic acid moiety and *m*/*z* 179 and 163 for the caffeic and *p*-coumaric acid moieties, supported their tentative annotation [23]. Esculetin (**2**), a hydroxycoumarin, was also detected, confirming the occurrence of phenolic metabolites with recognized antioxidant properties [24].

The extract also contained several flavonoid glycosides, including astragalin (**10**) and diosmetin-7-*O*-rutinoside (**11**), identified through the characteristic neutral loss of sugar moieties and the formation of diagnostic aglycone ions. In particular, astragalin (**10**) generated the kaempferol ion at *m*/*z* 285, whereas diosmetin-7-*O*-rutinoside (**11**) produced the diosmetin ion at *m*/*z* 299. Flavonoid glycosides are among the major specialized metabolites of the genus *Urtica* and are considered important contributors to its antioxidant and anti-inflammatory activities [25].

In addition to phenolic constituents, several compounds were tentatively assigned as terpenoid glycosides, including two putative terpenoid *O*-hexosides (**7** and **8**), an oxygenated terpenoid glycoside (**12**), and two putative geranylphenylacetate glycosides (**13** and **14**), suggesting the occurrence of glycosylated terpenoid–phenylpropanoid conjugates [26].

A remarkable feature of the metabolomic profile was the abundance of oxidized fatty acids and oxylipins. Hydroxy-, oxo-, and trihydroxylated octadecanoid derivatives were detected, including 9,12,13-trihydroxy-10,15-octadecadienoic acid (**19**), trihydroxyoctadecenoic acid (**21**), linolenic acid 13-hydroperoxide isomers (**23**, **25**, and **26**), 9,12-dihydroxy-10-octadecenoic acid (**27**), hydroxy- and oxo-octadecadienoic acid isomers (**29**, **30**, **33**, **34**, and **36**), a phytoprostane derivative (**24**), and a phytofuran isomer (**16**). These metabolites originate from the oxidation of polyunsaturated fatty acids through enzymatic and non-enzymatic pathways and are recognized as important mediators of plant responses to oxidative and environmental stress [27].

The extract also contained several aliphatic dicarboxylic acids, including azelaic (**9**), sebacic (**17**), undecanedioic (**20**), dodecanedioic (**22**), and oxododecanedioic (**15**) acids. Among these, azelaic acid (**9**) is particularly noteworthy because of its reported involvement in systemic acquired resistance and defense signaling in plants [28,29].

Overall, the phytochemical profile of the UUEE revealed a chemically diverse metabolome dominated by phenolic compounds and oxidized lipid derivatives. The coexistence of flavonoid glycosides, hydroxycinnamic acids, terpenoid glycosides, and oxylipins provides a plausible chemical basis for the reported antioxidant and anti-inflammatory properties of this species.

### 2.2. Antioxidant Activity of UUEE

As shown in Table 2, UUEE exhibited measurable antioxidant activity across the tested assays. The extract showed DPPH and ABTS radical-scavenging activities, with IC_50_ values of 445.10 ± 7.27 µg/mL and 212.49 ± 8.91 µg/mL, respectively. In the CUPRAC assay, UUEE showed reducing activity, with a value of 166.46 ± 6.49 µg/mL. However, these values were substantially higher than those recorded for the synthetic antioxidant standards BHA and BHT, indicating that UUEE had lower antioxidant potency than the reference compounds. Among the tested assays, UUEE showed greater activity against the ABTS radical cation than against the DPPH radical, as reflected by its lower IC_50_ value.

### 2.3. Total Phenolic and Total Flavonoid Contents

The total phenolic and flavonoid content of UUEE were found to be 16.86 ± 3.92 mg of gallic acid equivalent (GAE)/g of dry weight (DW) and 2.46 ± 0.01 mg of rutin equivalent (RE)/g, respectively (Table 3).

### 2.4. In Vitro Anti-Arthritic Effect

**Figure 1:**
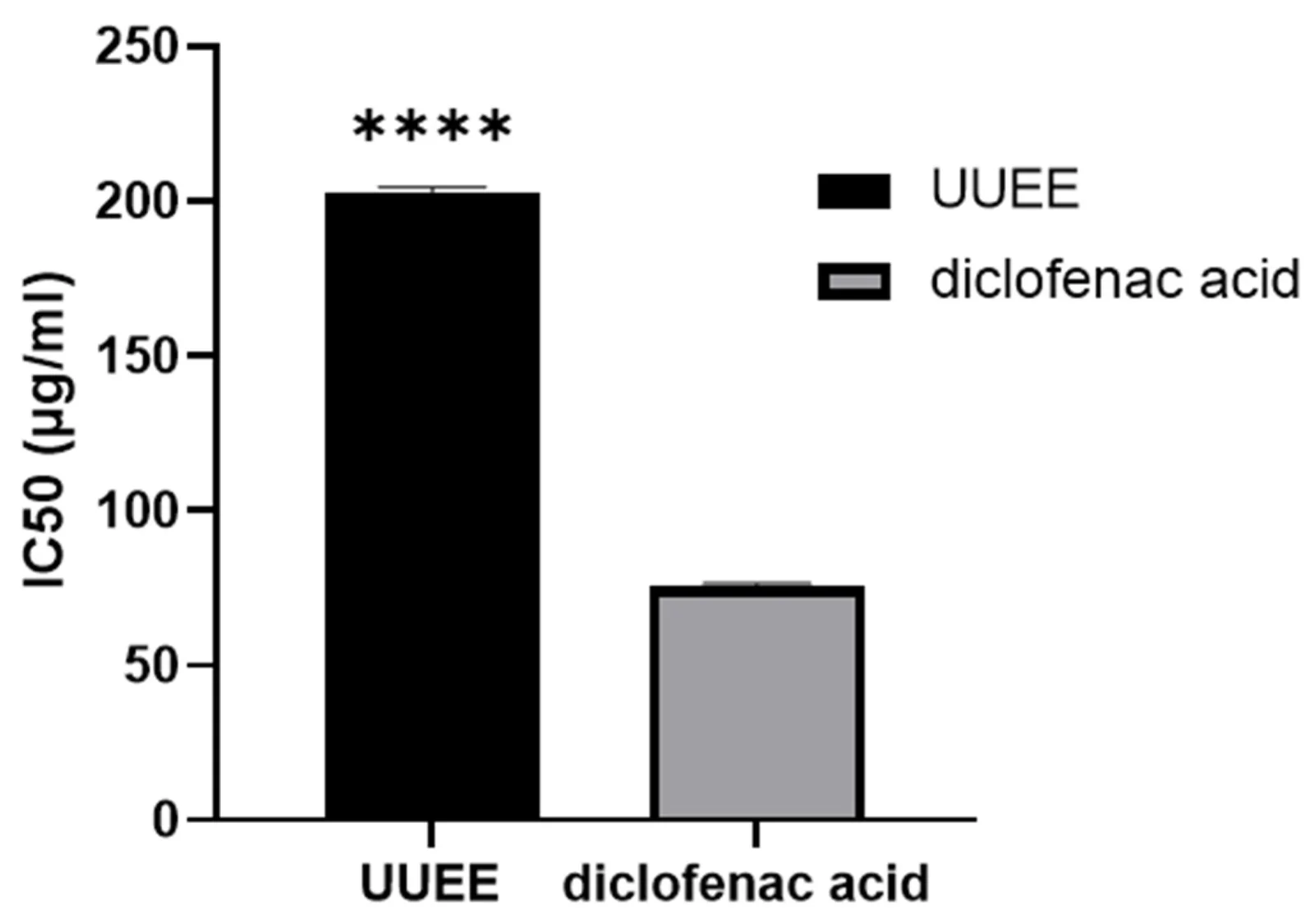
The in vitro anti-arthritic activity of UUEE compared with diclofenac acid. Values represent mean ± SD (*n* = 3). Statistical analysis was performed using an unpaired two-tailed Student’s *t*-test. **** *p* < 0.0001.

The UUEE significantly inhibited heat-induced denaturation of bovine serum albumin in a concentration-dependent manner (Figure 1). UUEE demonstrated measurable inhibition of protein denaturation in vitro, with an IC_50_ value of 202.54 ± 2.06 µg/mL. Nevertheless, diclofenac acid exhibited significantly greater inhibitory potency, with an IC_50_ of 75.57 ± 0.83 µg/mL. Thus, UUEE showed moderate protein-denaturation inhibitory activity, but its activity was lower than that of diclofenac acid.

### 2.5. In Vitro Cytotoxicity Effect

**Figure 2:**
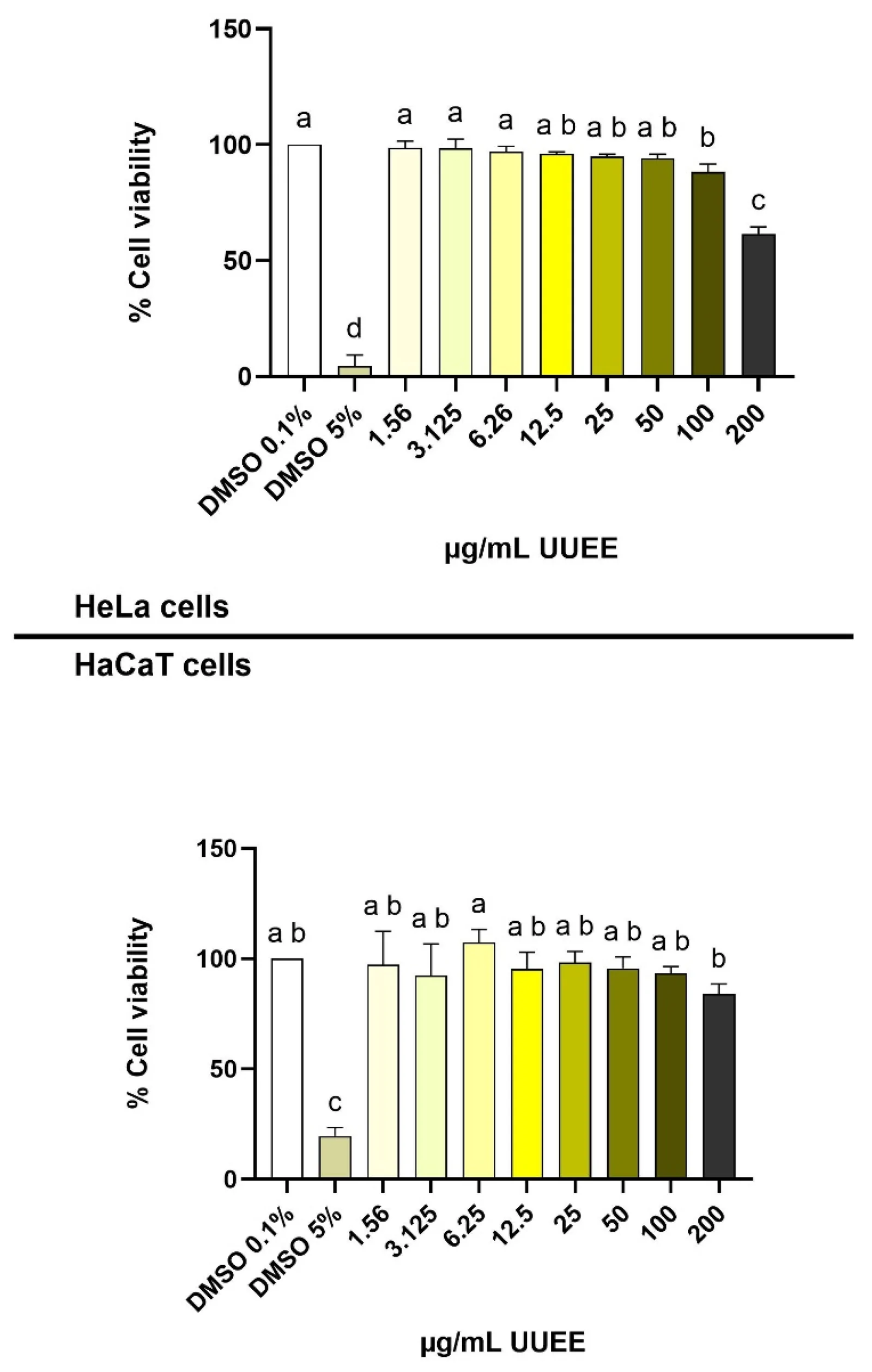
Cytotoxic effects of UUEE on HeLa and HaCaT cells after 24 h of treatment. Cells were treated with increasing concentrations (1.56–200 μg/mL) of the extract. Cell viability is expressed as the percentage of the vehicle-treated control (0.1% DMSO). Values are reported as the mean ± SD (*n* = 3). Statistical significance was determined by one-way ANOVA followed by Tukey’s multiple comparisons test. Bars with different lowercase letters differ significantly (*p* < 0.05).

The cytotoxic activity of the UUEE was investigated in HeLa and HaCaT cells following 24 h of exposure using the CCK-8 assay. Cells were treated with increasing concentrations of the extract (1.56–200 μg/mL), and cell viability was expressed as a percentage relative to the vehicle-treated control (0.1% DMSO) (Figure 2).

In HeLa cells, treatment with the extract at concentrations ranging from 1.56 to 100 μg/mL did not induce a marked reduction in cell viability, which remained close to the control values throughout this concentration range. Although a slight concentration-dependent decrease was observed between 25 and 100 μg/mL, these changes were not statistically significant compared with the vehicle control. In contrast, exposure to 200 μg/mL significantly reduced cell viability to approximately 60%, indicating a cytotoxic effect at the highest concentration tested (*p* < 0.05). As expected, treatment with 5% DMSO, used as the positive control, caused a pronounced decrease in cell viability, confirming the responsiveness of the assay. A comparable pattern was observed in HaCaT cells. Cell viability remained above 90% following treatment with 1.56–100 μg/mL of the extract, with no statistically significant differences relative to the vehicle-treated control. At 200 μg/mL, a modest reduction in viability was detected; however, cell survival remained above 80%, suggesting that the extract exerted only a limited cytotoxic effect on HaCaT cells at this concentration. As observed for HeLa cells, exposure to the positive control (5% DMSO) resulted in a significant reduction in cell viability.

Overall, the UUEE exhibited low cytotoxicity in both cell lines at concentrations up to 100 μg/mL. A significant reduction in cell viability was detected only at the highest concentration tested (200 μg/mL), with the effect being more evident in HeLa cells than in HaCaT cells.

### 2.6. In Vitro Genotoxicity Activity

**Figure 3:**
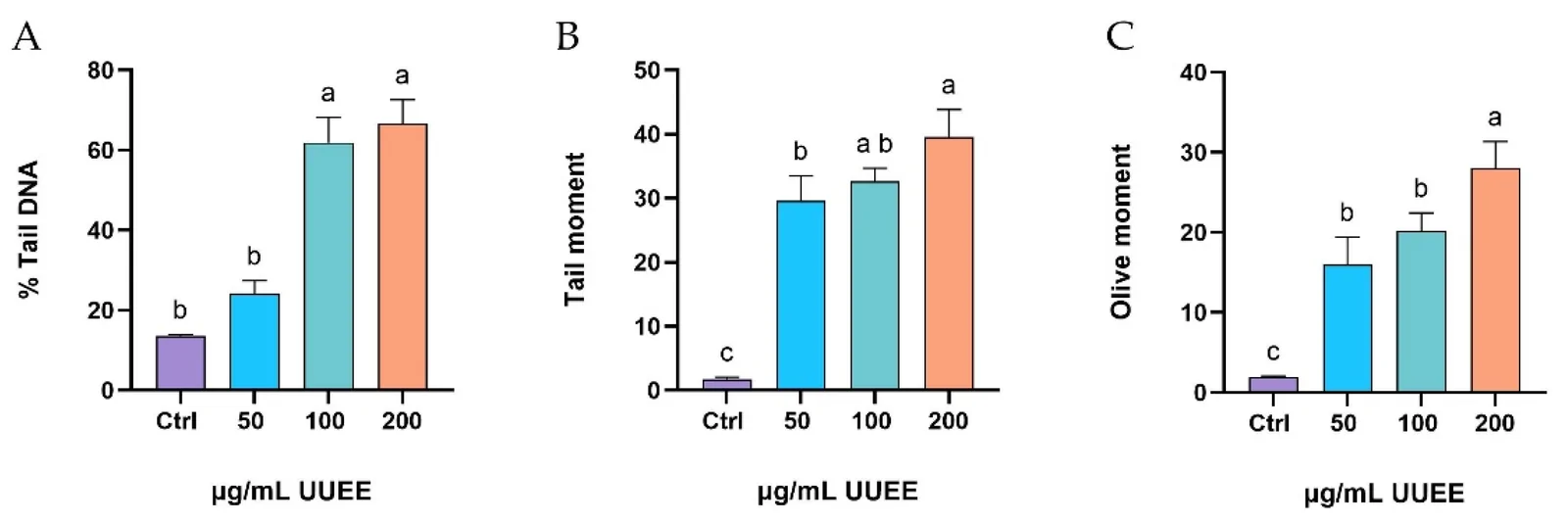
Effect of UUEE on DNA damage in HeLa cells determined by the alkaline comet assay after 24 h of treatment. DNA damage was quantified as (**A**) percentage of DNA in the tail, (**B**) tail moment, and (**C**) Olive tail moment following exposure to 50, 100, and 200 μg/mL of the extract. Values are reported as the mean ± SD (*n* = 3). Statistical significance was determined by one-way ANOVA followed by Tukey’s multiple comparisons test. Different lowercase letters denote significant differences among groups (*p* < 0.05).

The genotoxic potential of the UUEE was evaluated in HeLa cells using the alkaline comet assay after 24 h of treatment. DNA damage was quantified by measuring the percentage of DNA in the tail, tail moment, and Olive tail moment (Figure 3).

As shown in Figure 3A, treatment with 50 μg/mL of the extract induced a significant increase in the percentage of tail DNA compared to the untreated control (*p* < 0.05). Higher concentrations of 100 and 200 μg/mL produced a further significant elevation in this parameter relative to both the control and the 50 μg/mL treatment group. Notably, no statistically significant difference was observed between the 100 and 200 μg/mL groups.

For the tail moment (Figure 3B), exposure to 50 and 100 μg/mL resulted in significantly higher values compared to those measured in the control group, with no significant differences between these two concentrations. The 200 μg/mL treatment yielded the most pronounced increase, which was significantly greater than both the control and the lower concentration groups (*p* < 0.05).

Similarly, the Olive tail moment (Figure 3C) increased in a concentration-dependent manner following UUEE treatment. Both 50 and 100 μg/mL significantly elevated this parameter relative to the untreated control, whereas the highest value was recorded at 200 μg/mL, which differed significantly from all other experimental groups (*p* < 0.05).

Collectively, these results indicate that UUEE induces concentration-dependent DNA damage in HeLa cells, with the most marked effects observed at the highest tested concentration of 200 μg/mL.

### 2.7. Effects of UUEE on Zymosan-Induced IL-1β and IL-6 Gene Expression

**Figure 4:**
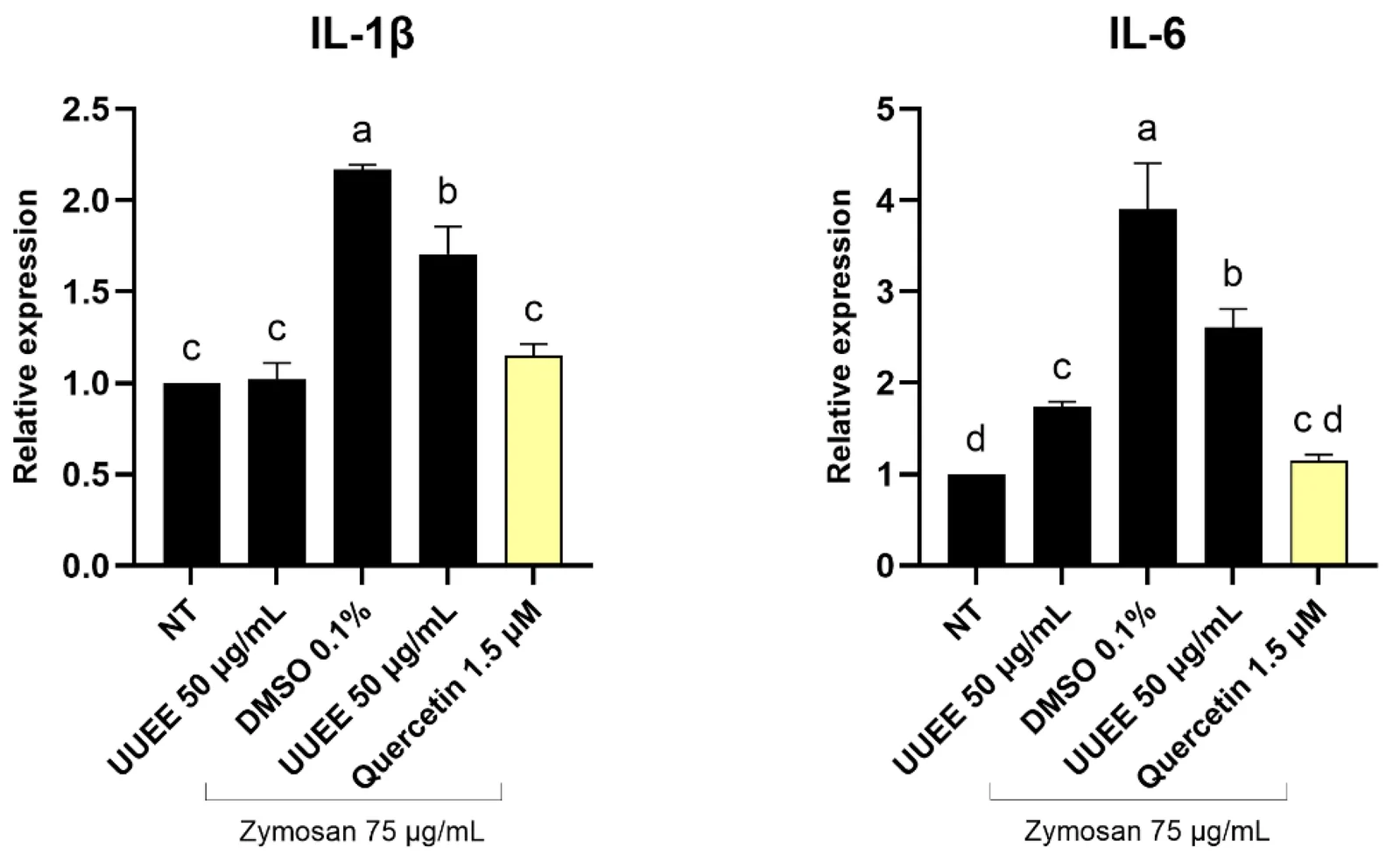
Effect of UUEE on IL-1β and IL-6 gene expression in zymosan-stimulated cells. Cells were treated with zymosan (75 μg/mL) in the presence or absence of the extract. Gene expression levels were normalized to a housekeeping gene (GAPDH) and expressed as fold change relative to the control. Different letters indicate statistically significant differences (*p* < 0.05) between groups, as determined by one-way ANOVA followed by Tukey’s post hoc test. Data are presented as mean ± SD (*n* = 3).

Stimulation with zymosan (75 μg/mL) significantly increased the expression of the pro-inflammatory interleukins IL-1β and IL-6 compared to untreated cells (Figure 4). Pretreatment with UUEE (50 μg/mL) followed by stimulation with zymosan significantly reduced the expression of both genes, compared to values observed in cells pretreated with 0.1% DMSO. These results indicate that UUEE modulates zymosan-induced IL-1β and IL-6 gene expression in HaCaT cells under the experimental conditions used.

### 2.8. Effect of UUEE Against CFA Induced Arthritis in Wistar Rats

#### 2.8.1. Effect on Paw, Joint Thickness, Arthritis Score and Body Weight

**Figure 5:**
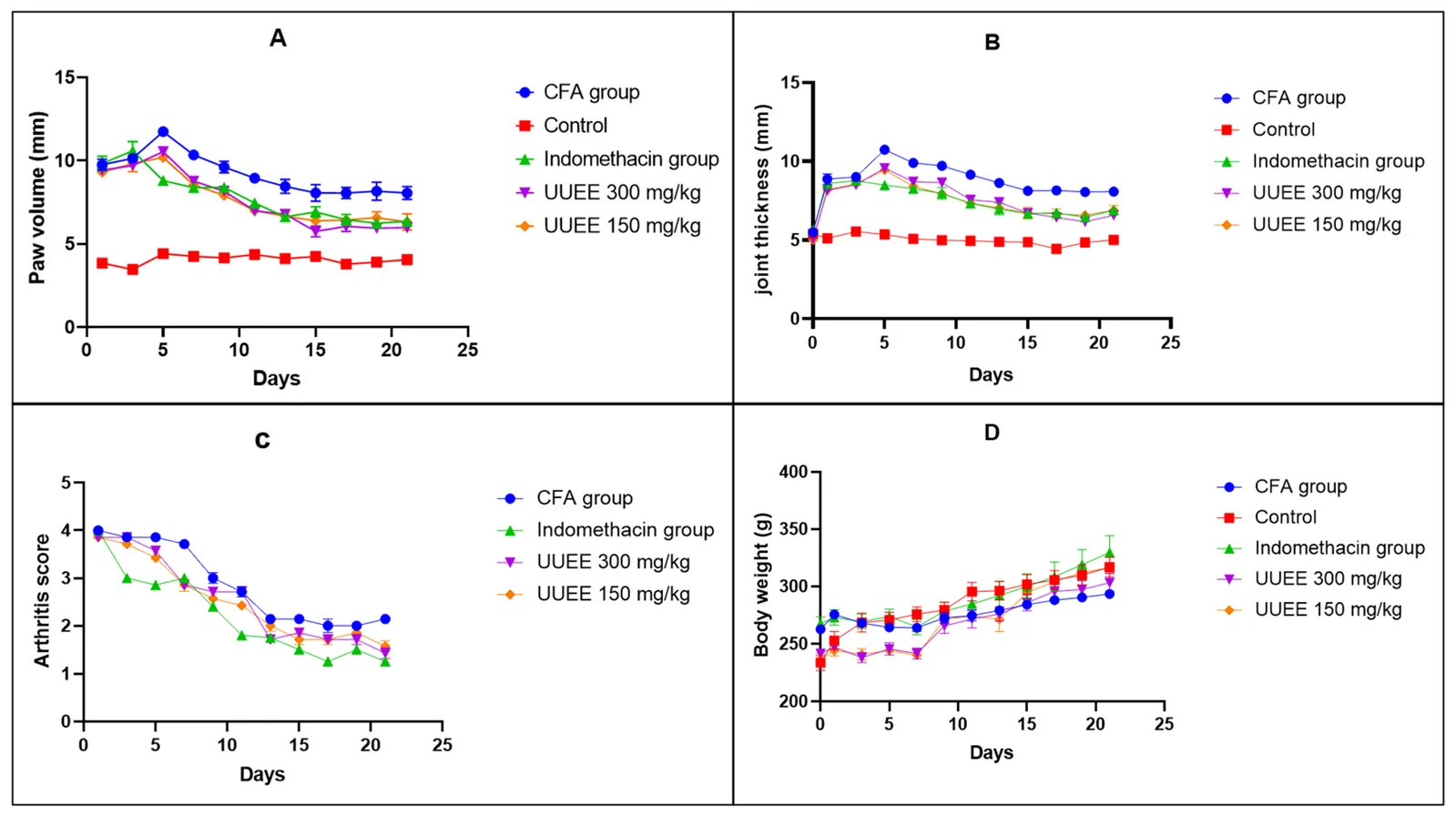
Effect of UUEE (150 and 300 mg/kg, p.o.) and indomethacin on (**A**) paw volume, (**B**) joint thickness, (**C**) arthritis score, and (**D**) body weight in CFA-induced arthritic Wistar rats. Values are expressed as mean ± SEM (*n* = 7). *p* < 0.0001 vs. CFA group (two-way ANOVA followed by Tukey’s multiple comparisons test).

As shown in Figure 5A,B rats injected with CFA exhibited a significant increase in paw and joint thickness (*p* < 0.0001) compared to the control group, with peak inflammation observed on day 3 post-induction. Treatment with UUEE at 300 mg/kg led to a marked reduction in both paw and joint swelling (*p* < 0.0001 vs. CFA group), while the 150 mg/kg dose showed moderate efficacy. Indomethacin (5 mg/kg) also significantly reduced inflammation (*p* < 0.0001 vs. CFA group). By day 21, UUEE at 300 mg/kg reduced paw thickness to 5.94 ± 0.15 mm and joint thickness to 6.87 ± 0.15 mm, while 150 mg/kg achieved 6.31 ± 0.48 mm (paw) and 6.89 ± 0.3 mm (joint), respectively. In comparison, the CFA group maintained markedly elevated values of 8.05 ± 0.37 mm (paw) and 8.08 ± 0.13 mm (joint).

Arthritis scores increased rapidly after CFA injection and remained elevated in the CFA group, indicating severe joint inflammation (Figure 5C). In contrast, rats treated with UUEE showed a gradual and consistent decrease in arthritis scores, with the 300 mg/kg dose producing the most pronounced improvement, closely matching the reference drug (**** *p* < 0.0001 vs. CFA).

Body weight gain was markedly suppressed in CFA-induced arthritic rats compared with controls, reflecting disease-associated cachexia (Figure 5D). Oral administration of UUEE significantly improved body weight from the second week onward, particularly at 300 mg/kg, indicating attenuation of systemic illness and an overall improvement in health status comparable to that observed in the indomethacin group (**** *p* < 0.0001 vs. CFA group).

#### 2.8.2. Effect of UUEE on Oxidative Stress Markers in Liver Homogenate

**Figure 6:**
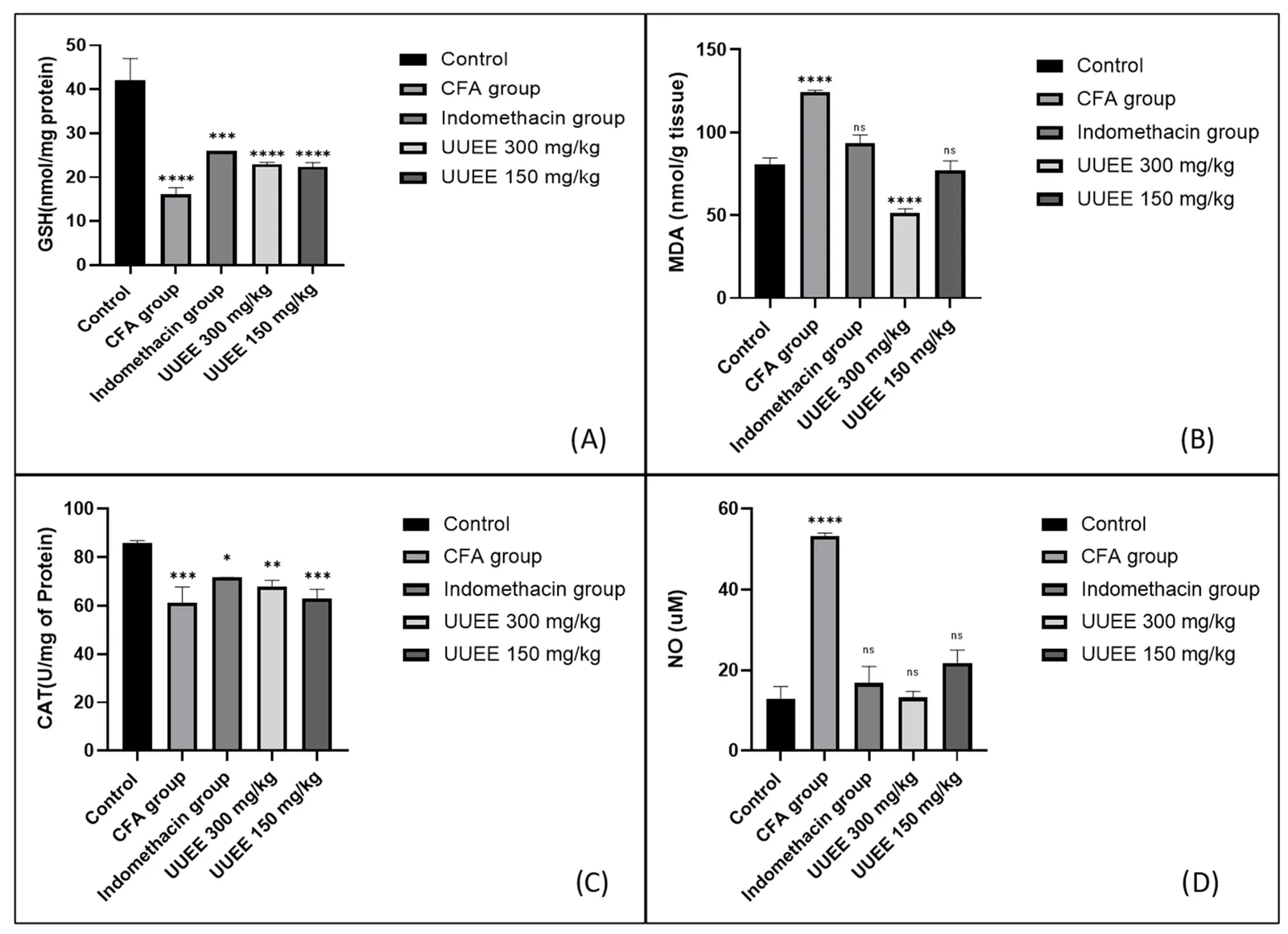
Effect of UUEE (150, 300 mg/kg) and indomethacin on (**A**) GSH, (**B**) MDA, (**C**) CAT, and (**D**) NO levels in liver homogenate of CFA-induced arthritic rats. Values are mean ± SEM (*n* = 7). Statistical significance was determined by one-way ANOVA followed by Dunnett’s multiple comparisons test. * *p* < 0.05, ** *p* < 0.01, *** *p* < 0.001, **** *p* < 0.0001; ns indicates non-significant differences vs. control group.

CFA administration significantly increased MDA and NO levels while decreasing GSH and CAT activity in liver homogenate compared with the control group (Figure 6).

GSH content was significantly reduced in CFA-treated rats (*p* < 0.0001) versus control groups. UUEE (150 mg/kg, 300 mg/kg) and indomethacin significantly restored GSH levels compared to the CFA group. CAT activity decreased in the CFA group (*p* < 0.001). UUEE (150 mg/kg, 300 mg/kg) and indomethacin significantly elevated CAT activity.

MDA levels were markedly higher in CFA-treated rats (*p* < 0.0001) versus normal control group; however, UUEE (300 mg/kg) significantly reduced liver MDA compared to the CFA group. NO levels were markedly increased in the CFA group (*p* < 0.0001). Treatment with UUEE at 150 mg/kg and 300 mg/kg, as well as indomethacin, caused a non-significant reduction in NO production compared with control group.

#### 2.8.3. Effect of UUEE on Oxidative Stress Markers in Spleen Homogenate

**Figure 7:**
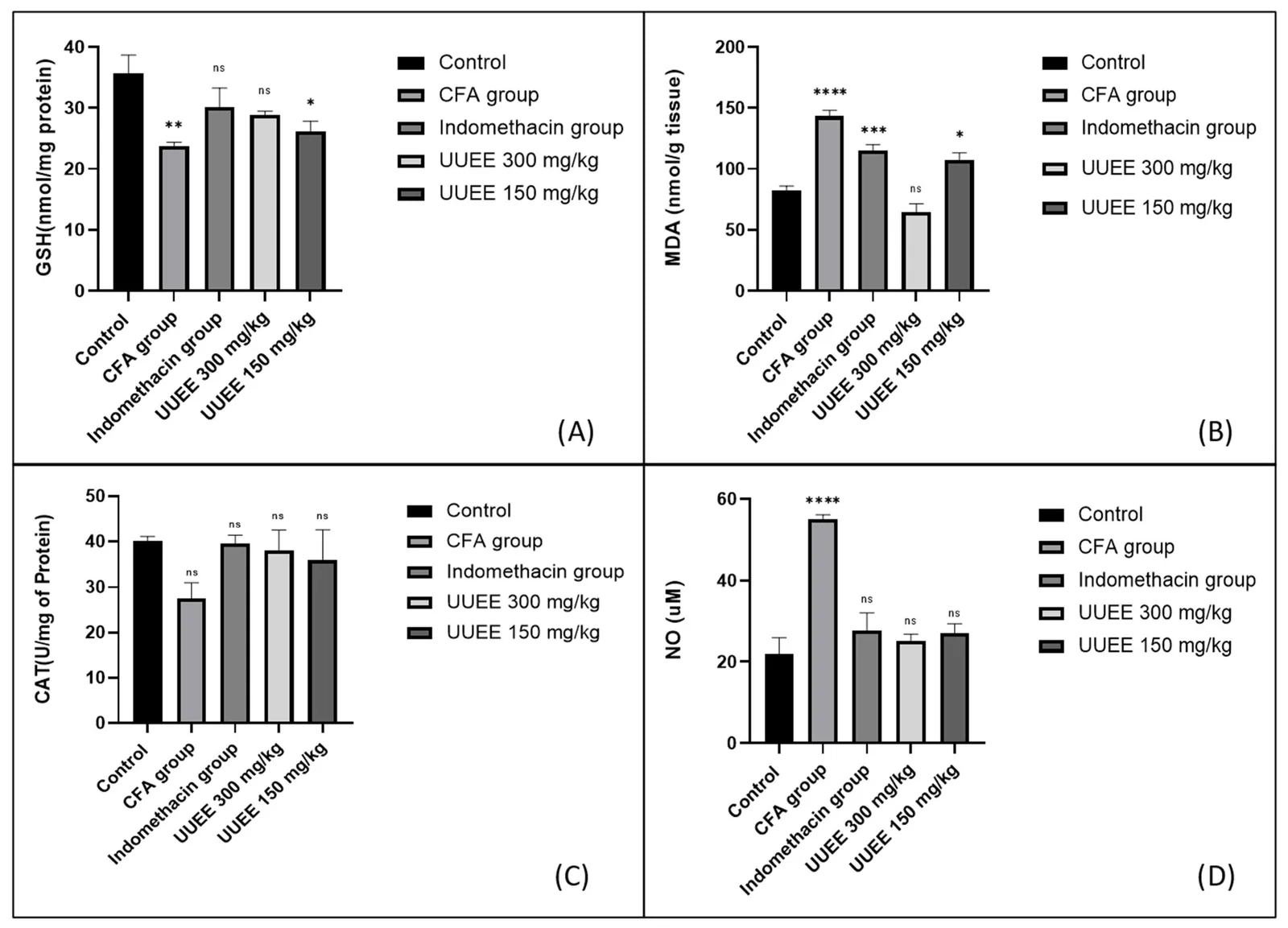
Effect of UUEE (150, 300 mg/kg) and indomethacin on (**A**) GSH, (**B**) MDA, (**C**) CAT, and (**D**) NO levels in spleen homogenate of CFA-induced arthritic rats. Values are mean ± SEM (*n* = 7). Statistical significance was determined by one-way ANOVA followed by Dunnett’s multiple comparisons test * *p* < 0.05, ** *p* < 0.01, *** *p* < 0.001, **** *p* < 0.0001; ns indicates non-significant differences vs. control group.

CFA administration significantly increased MDA and NO levels while decreasing GSH and CAT activity in spleen homogenate compared with the control group (Figure 7).

GSH content was significantly reduced in CFA-treated rats (*p* < 0.01) versus normal control group. UUEE (150 mg/kg, 300 mg/kg) and indomethacin significantly restored GSH levels compared to the CFA group. CAT activity decreased in the CFA group. UUEE (150 mg/kg, 300 mg/kg) and indomethacin significantly elevated CAT activity.

MDA levels were markedly higher in CFA-treated rats (*p* < 0.0001) versus control groups; however, UUEE (150 mg/kg, 300 mg/kg) and indomethacin significantly reduced splenic MDA compared to the CFA group. NO levels were substantially elevated in the CFA group (*p* < 0.0001). Treatment with UUEE (150 mg/kg, 300 mg/kg) and indomethacin significantly lowered NO production relative to CFA-treated rats.

#### 2.8.4. Effect on Hematological and Biochemical Parameters

The blood and serum profile of rats treated with UUEE and indomethacin is given in Table 4. The fall in RBCs and HGB, whereas an upswing in WBCs and platelets was observed in CFA group rats. No such changes were noted in the normal control group. Likewise, treatment with UUEE (150 and 300 mg/kg) and indomethacin prevented abnormal alterations in hematological values altered by adjuvant injection.

CFA induction significantly increased ASAT activity. UUEE treatment at 300 mg/kg significantly decreased both ASAT and ALAT activities, whereas the 150 mg/kg dose significantly decreased ALAT only. Indomethacin produced numerical reductions in ASAT and ALAT compared with the CFA group, but these changes were not statistically significant. Urea and creatinine levels were also numerically lower in the treated groups; however, the differences did not reach statistical significance.

#### 2.8.5. Histopathological Findings

**Figure 8:**
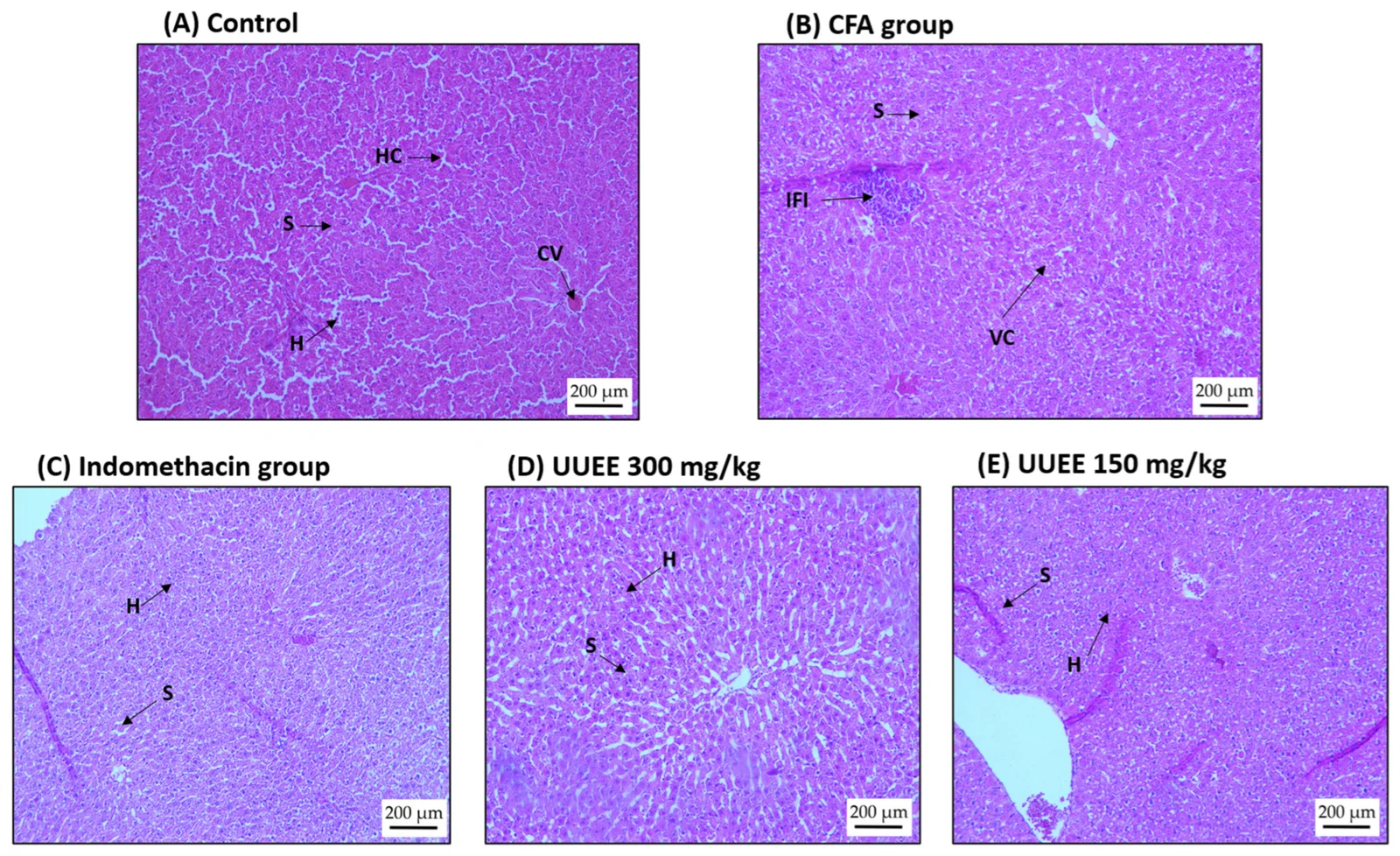
Histopathological examination (×20) of rat livers. (**A**) Normal liver tissue. (**B**) Liver tissue of the CFA-induced arthritic group. (**C**) Liver tissue from rats treated with indomethacin (5 mg/kg). (**D**) Liver tissue from rats treated with UUEE (300 mg/kg). (**E**) Liver tissue from rats treated with UUEE (150 mg/kg). CV: central vein; H: hepatocytes; HC: hepatic cords; S: sinusoids; VC: vascular congestion; IFI: inflammatory cell infiltration.

Histologically, the doses of UUEE used (150 and 300 mg/kg) exhibited no signs of toxicity in the liver as evidenced by the absence of tissue damage, necrosis, or structural alterations compared to the control group (Figure 8). The only minor observation was slight, non-pathological vascular congestion and focal inflammatory infiltrates noted in CFA-induced arthritic rats.

**Figure 9:**
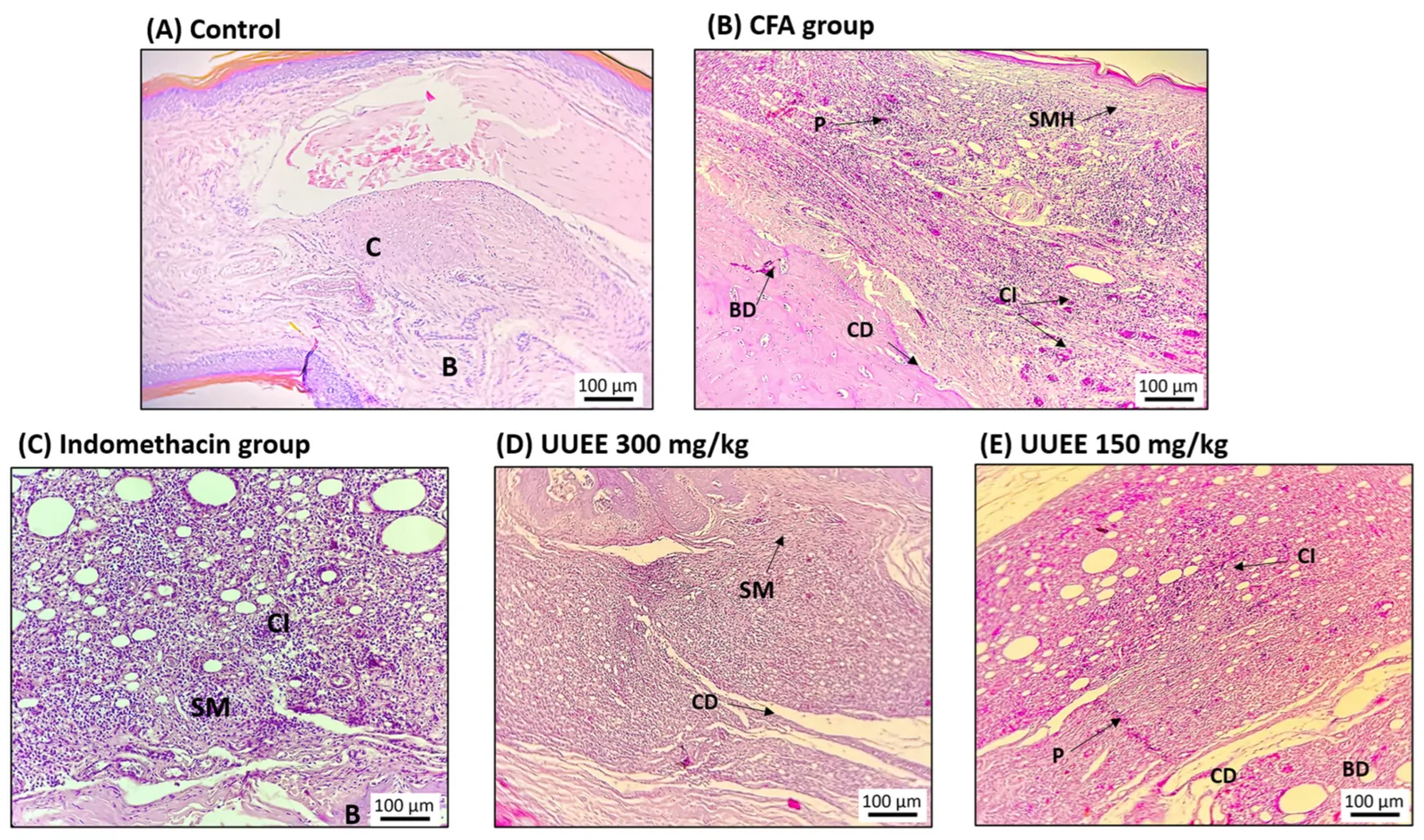
Histopathological examination (×10) of paws from CFA-induced arthritic rats. (**A**) Normal control showing normal joint architecture with a thin synovial lining, intact cartilage and bone, and no inflammatory infiltration, and no synovial pannus formation. (**B**) CFA group showing synovial membrane hyperplasia, dense inflammatory cell infiltration, cartilage destruction, and bone destruction. (**C**) Indomethacin group showing marked attenuation of inflammatory changes with preservation of joint architecture. (**D**) UUEE 300 mg/kg group showing only mild synovial hyperplasia and minimal inflammatory infiltration with preservation of cartilage and bone (**E**) UUEE 150 mg/kg group showing moderate synovial hyperplasia and inflammatory infiltration with partial cartilage preservation. SM: synovial membrane; SMH: synovial membrane hyperplasia; P: pannus; CI: cell infiltration; C: cartilage; CD: cartilage destruction; B: bone; BD: bone destruction.

Histopathological examination of paws revealed marked structural alterations in CFA-induced arthritic rats compared with control groups. Joints from the CFA group showed extensive synovial hyperplasia with thickening of the synovial membrane, dense inflammatory cell infiltration, pannus formation invading the articular surface, and evident cartilage and subchondral bone erosion (Figure 9). In contrast, normal control rats exhibited a preserved joint architecture with a thin synovial lining, intact cartilage, and normal bone structure without inflammatory infiltrates. Treatment with UUEE attenuated these CFA-induced histopathological changes in a dose-dependent manner. In the UUEE 150 mg/kg group, a moderate reduction in synovial proliferation and inflammatory infiltration was observed, with partial preservation of cartilage integrity. Joints from rats treated with UUEE 300 mg/kg displayed only mild synovial thickening and minimal pannus formation, while cartilage and bone structures appeared largely preserved, approaching the morphology seen in the indomethacin-treated group.

## 3. Discussion

RA is a chronic inflammatory disorder that typically involves multiple joints symmetrically, especially the small joints of the hands and feet, while current treatment options are available, they often carry significant side effects, highlighting the need for safer and more widely effective alternatives [30,31]. In recent years, there has been growing interest in herbal remedies for their potential to help prevent and manage a range of health conditions, including RA [31]. *U. urens*, which belongs to the family of Urticaceae and commonly known as nettle has been used extensively as a traditional medicine in many countries for the treatment of anemia, rheumatism and arthritis, eczema, asthma, urinary gravel, stomach complaints, skin infections and as an anti-haemorrhagic [16,32]. In the present study, we have established for the first time that the UUEE leaves effectively alleviated rheumatoid arthritis in both in vitro and in vivo arthritis models.

Antioxidant potentiality of UUEE was investigated using DPPH, ABTS and CUPRAC assays, Reactive oxygen species (ROS) and free radicals are highly reactive and unstable molecules that can exert harmful effects on biological systems by damaging DNA, proteins, and lipids [33]. Antioxidants, on the other hand, help stabilize or neutralize these free radicals. Various antioxidant compounds found in medicinal plants such as phenolics, tannins, flavonoids, proanthocyanidins, lignans, terpenoids, stilbenes, coumarins, quinones, alkaloids, and other metabolites have been reported to play a protective role against diseases associated with ROS and oxidative stress [34]. The present study demonstrated that UUEE exhibited appreciable antioxidant activity in all in vitro antioxidant assays; however, its antioxidant potency was lower than that of the standard antioxidants, BHA and BHT. This activity may be linked to the phytochemical composition of the extract, which was found to be rich in polyphenols and flavonoids, these compounds are well known for their free radical scavenging abilities and redox properties, suggesting that the antioxidant potential of *U. urens* is largely attributable to the presence of these bioactive constituents [33,34,35].

Protein denaturation is a well-recognized contributing factor in the development of RA [36]. Among the key mediators of RA progression are matrix metalloproteases (MMPs), particularly MMP-2 and MMP-9 (gelatinases), which play a crucial role in cartilage degradation by breaking down essential extracellular matrix components such as type II collagen and proteoglycans, their expression and activity are largely driven by pro-inflammatory cytokines and oxidative stress, creating a vicious cycle of ongoing inflammation and tissue destruction, therefore targeting and inhibiting MMPs may help slow joint degeneration and provide protective effects against RA-related damage [37]. In the current investigation, the *U. urens* extract significantly inhibited heat-induced protein denaturation using BSA as a model protein. Based on these findings, it can be inferred that the ability of *U. urens* to prevent protein denaturation and thereby reduce auto-antigen generation may play a key role in its observed anti-arthritic activity.

UUEE exerted only limited cytotoxic effects on both HeLa and HaCaT cells after 24 h of treatment. Cell viability remained above 90% at concentrations up to 100 μg/mL, whereas a significant reduction was observed only at the highest concentration tested (200 μg/mL), particularly in HeLa cells. These findings indicate that UUEE has limited effects on short-term cell viability at concentrations up to 100 μg/mL. However, cell viability alone cannot be considered sufficient to establish the overall safety of the extract, particularly with regard to its potential effects on genomic integrity.

Species belonging to the genus *Urtica* are generally recognized as safe and have long been used in traditional medicine for the treatment of inflammatory disorders [38,39]. Previous studies on *Urtica dioica* and other *Urtica* species have reported low cytotoxicity toward normal mammalian cells at moderate concentrations, while higher doses may reduce the viability of tumor cell lines through mechanisms involving oxidative imbalance, mitochondrial dysfunction, and apoptosis [13]. Nevertheless, these observations cannot be directly extrapolated to the genomic safety of the UUEE under investigation in this study. The moderate reduction in HeLa cell viability observed at 200 μg/mL in the present study may therefore reflect the onset of concentration-dependent cytotoxic mechanisms. Considering the phytochemical profile of the extract, particularly its richness in phenolic acids and flavonoids, the observed biological activity may result from the combined effects of multiple bioactive compounds rather than from a single constituent [40,41].

Inflammatory interleukins such as IL-1β and IL-6 play central roles in the initiation and progression of rheumatoid arthritis by promoting synovial inflammation, leukocyte recruitment, cartilage degradation, and osteoclast-mediated bone resorption [42]. Consequently, the inhibition of these inflammatory interleukins represents a major therapeutic target for limiting disease progression [42]. In the present study, zymosan stimulation markedly increased the expression of both IL-1β and IL-6, confirming the establishment of a robust inflammatory response. Pretreatment with UUEE significantly attenuated the expression of both interleukins, demonstrating its ability to modulate the inflammatory response at the transcriptional level. These findings suggest that the anti-inflammatory activity of UUEE may be, at least in part, attributed to the suppression of key pro-inflammatory cytokines involved in the amplification of inflammatory signaling. Considering the phytochemical profile of the extract, particularly its high content of phenolic acids and flavonoids, the observed effect is likely the result of the synergistic action of multiple bioactive constituents, which have been widely reported to interfere with inflammatory pathways such as NF-κB and MAPK signaling, ultimately reducing cytokine production [43]. In the present study, zymosan stimulation markedly increased the expression of both interleukins, confirming the establishment of an inflammatory response in HaCaT cells. Pretreatment with UUEE significantly attenuated the expression of IL-1β and IL-6, indicating that the extract can modulate cytokine expression under the experimental conditions employed. Considering the phytochemical profile of the extract, particularly the presence of phenolic acids and flavonoids, this effect may result from the combined activity of multiple bioactive constituents that have been reported to modulate inflammatory signaling pathways [43].

However, the relevance of the HaCaT keratinocyte model to rheumatoid arthritis should be interpreted with caution. Although HaCaT cells provide a reproducible in vitro model for investigating epithelial inflammatory responses, they do not directly represent the major cellular populations involved in rheumatoid joint inflammation [44]. Therefore, the observed downregulation of IL-1β and IL-6 should be considered evidence of an immunomodulatory effect in this specific cellular model rather than direct evidence of anti-inflammatory activity in synovial tissue. Further studies using more disease-relevant models, including macrophage/monocyte-derived cells, synovial fibroblasts, and primary human cells, will be necessary to better define the mechanisms underlying the anti-inflammatory activity of UUEE in the context of rheumatoid arthritis.

The alkaline comet assay revealed a concentration-dependent increase in DNA damage in HeLa cells following treatment with the UUEE. All three comet parameters (% tail DNA, tail moment, and Olive moment) increased with extract concentration, with the most pronounced effects observed at 200 μg/mL. Importantly, a statistically significant increase in DNA damage was already observed at 50 μg/mL, indicating that genotoxic effects can occur at concentrations that do not produce a significant reduction in cell viability. Since the comet assay detects DNA single-strand breaks, alkali-labile sites, and transient repair intermediates, these results indicate that exposure to high concentrations of the extract affects DNA integrity [45].

The present findings clearly demonstrate that cytotoxicity and genotoxicity do not necessarily occur at the same concentration range. Although a significant reduction in cell viability was observed mainly at 200 μg/mL, the comet assay revealed significant DNA damage starting at 50 μg/mL. Therefore, the absence of a marked reduction in cell viability at 50 and 100 μg/mL cannot be interpreted as evidence of genomic safety. Cell viability and DNA integrity represent distinct biological endpoints, and cells may retain metabolic activity despite the presence of DNA damage [46]. It should also be considered that several plant-derived polyphenols exhibit a dual biological behaviour [47]. Although they are widely recognized for their antioxidant properties at low and moderate concentrations, under specific experimental conditions they may exert pro-oxidant effects capable of inducing oxidative DNA damage, particularly in rapidly proliferating cancer cells. This concentration-dependent biphasic activity has been described for numerous flavonoids and phenolic compounds.

Because the comet assay was performed in HeLa cells, a highly proliferative cancer cell line, the observed DNA damage may be associated with an antiproliferative or pro-apoptotic response. However, the present results do not allow us to distinguish between potentially reversible DNA lesions, oxidative DNA damage, DNA repair intermediates, or irreversible genomic injury. Therefore, the observed genotoxic effects should not be interpreted as beneficial or selective anticancer activity without further mechanistic evidence.

Indeed, controlled induction of DNA damage is a common mechanism through which several natural compounds suppress tumor cell growth by activating DNA damage response pathways, cell-cycle arrest, and apoptosis [48]. Nevertheless, additional investigations, including the assessment of apoptotic markers, intracellular ROS production, and DNA repair pathways, are required to clarify whether the DNA damage observed in the present study represents reversible oxidative lesions or irreversible genomic injury leading to cell death.

Overall, the combined cytotoxicity and comet assay results indicate that UUEE exerts a concentration-dependent biological response. While concentrations up to 100 μg/mL produced limited effects on short-term cell viability, significant DNA damage was detected in HeLa cells starting at 50 μg/mL. Therefore, the present findings do not allow the concentrations tested to be considered unequivocally genomically safe. Further studies in non-cancerous human cells and in the relevant target cell models are necessary to define the safety profile of UUEE and to determine whether the observed genotoxic effects are cell-type specific.

The CFA-induced arthritis model in rats is effective in mimicking the immunological and pathological features of RA in people, making it a valuable tool for researchers [49,50].

In this research, we explored the effect of UUEE on rheumatoid arthritis (RA) induced by CFA in rats. Treatment with UUEE at doses of 150 and 300 mg/kg demonstrated a clear anti-arthritic effect across various signs of inflammation. Compared to the arthritic control group, UUEE significantly reduced inflammation, which was evident from the noticeable decrease in paw swelling and arthritis score.

Swelling and paw scoring are commonly used indicators to assess the anti-arthritic potential of various drugs [51]. Paw edema is typically associated with increased fluid and protein leakage, enhanced vascular permeability, and cellular infiltration in the inflamed area [52].

In the present study, the arthritic control group showed persistent inflammation up to day 21, characterized by sustained edema and continuous cellular infiltration. In contrast, the treatment groups exhibited peak inflammation on day 3, followed by a gradual reduction. The ethanolic extract demonstrated a significant reduction in paw volume and diameter compared to the CFA control group. It also markedly lowered arthritis scores and protected against rheumatoid arthritis-related morphological changes by reducing inflammation and redness.

The rats exhibited noticeable weight loss the day after receiving the adjuvant injection. However, over the subsequent days, their body weight gradually recovered, returning to a normal growth trajectory. The decrease in body weight observed during inflammation may be attributed to impaired nutrient absorption in the intestines, compounded by reduced food intake [53]. This reduction in intake is likely a result of physical immobility and hyperalgesia, both of which can contribute to the development of rheumatoid cachexia [51,54]. The findings of the present study also suggest a strong correlation between the degree of inflammation and the observed loss of body weight. Therefore, the recovery of body weight in rats treated with UUEE may be attributed to improved nutrient absorption in the intestines.

Aminotransferase activity rises significantly in arthritic rats, serving as a reliable indicator of liver dysfunction, common complications of adjuvant-induced arthritis [55]. Elevated serum levels of AST and ALT are known to contribute to the production of biologically active inflammatory mediators, such as bradykinins, which play a key role in the inflammatory response [49], Administration of UUEE led to a reduction in AST and ALT levels, suggesting a protective effect. This decrease is likely linked to the compound’s anti-inflammatory properties.

Anemia is a common complication associated with RA, often accompanied by an increase in red blood cell (RBC) and platelet count, which reflects disease progression [56]. In our study, the hematological analysis showed that UUEE (150 and 300 mg/kg) treatment effectively restored RBC count, hemoglobin (HGB) levels, and platelet count, as supported by improvements in arthritic indicators. Previous findings suggest that the moderate elevation in white blood cell (WBC) count seen in arthritis is driven by IL-1–induced stimulation of colony-forming factors [57]. Meanwhile, the reduction in HGB levels is attributed to decreased erythropoietin production, reduced bone marrow responsiveness, and increased premature destruction of RBCs [57].

Lipid peroxidation is initiated when oxidants, particularly free radicals, target lipids with carbon double bonds, primarily polyunsaturated fatty acids (PUFAs) leading to the formation of lipid peroxyl radicals [58]. These radicals trigger a chain reaction that further oxidizes PUFAs, ultimately damaging cell membranes. One of the key mutagenic byproducts of this process is malondialdehyde (MDA) [59]. In our study, UUEE treatment significantly lowered MDA levels.

GSH reflect the endogenous defence against damage caused by ROS and organic peroxides as they act as an intracellular reductant in oxidation reduction processes, the decreased levels of GSH in the liver and the spleen of arthritic rats might be due to the excessive consumption of GSH by the system to defend oxidative damage [60]. The presence of GSH serves as a defensive response mechanism, counteracting oxidative damage by acting as a reductant in various redox reactions within the cell, GSH plays a vital role in maintaining cellular integrity and protecting tissue structure, reduced GSH levels are commonly linked to various pathological conditions, often resulting from its increased utilization during oxidative stress and cell damage [29]. In our study, the arthritic control group showed a significant decline in GSH levels, indicating oxidative imbalance. However, oral administration of UUEE effectively restored these antioxidant levels, likely by enhancing the scavenging of free radicals and mitigating oxidative stress.

Catalase plays a crucial role in detoxifying reactive oxygen species, particularly by breaking down superoxide anions and hydrogen peroxide (H_2_O_2_), thus protecting cells from oxidative damage. Although catalase activity tends to decrease in rheumatoid arthritis (RA) [61]. Its levels are often insufficient to provide effective defence against elevated H_2_O_2_ concentrations. The observed reduction in catalase activity in RA is likely due to enzyme inactivation by excess H_2_O_2_, indicating its significant role in the disease process and in managing oxidative stress [29]. Previous studies have established a strong association between RA and oxidative stress, marked by elevated lipid peroxidation, depleted GSH levels, and reduced catalase activity [4]. Consistent with these findings, our results demonstrated that treatment with UUEE (150 and 300 mg/kg) significantly lowered oxidative stress markers in rats with CFA-induced arthritis.

Histopathological analysis of paws from CFA-induced arthritic rats revealed severe pathological alterations in the untreated disease control group, including pronounced synovial hyperplasia, massive inflammatory cell infiltration (predominantly neutrophils and mononuclear cells), pannus formation, extensive cartilage erosion, and subchondral bone destruction with osteoclast activation, these changes align with the chronic inflammatory cascade in RA models, where CFA triggers persistent immune activation leading to joint deformity and functional impairment [62]. UUEE treatment (150 and 300 mg/kg) dose-dependently ameliorated these features: low-dose reduced synovial thickening and infiltration, while high-dose markedly restored near-normal architecture with minimal edema, preserved cartilage integrity, reduced pannus, and limited bone erosion. These protective effects corroborate UUEE’s anti-inflammatory and antioxidant mechanisms, suppressing pro-inflammatory mediators that drive histopathological progression in CFA arthritis. Overall, UUEE demonstrates RA-modifying potential akin to phytotherapeutics.

To support possible clinical translation, the effective animal doses were converted to human equivalent doses (HED) using the body surface area normalization method of Nair and Jacob [63]. Using a rat Km value of 7 and a human Km value of 37, the 150 mg/kg dose is equivalent to an HED of about 28.4 mg/kg, or roughly 1.7 g/day for a 60 kg adult, while the 300 mg/kg dose corresponds to about 56.8 mg/kg, or approximately 3.4 g/day. When the recommended safety factor of 10 for first-in-human studies is applied, the maximum recommended starting dose based on the more effective 150 mg/kg dose is about 2.84 mg/kg, or around 170 mg/day. However, HED estimates should be viewed as preliminary and do not account for differences between species in pharmacokinetics, metabolism, or oral bioavailability.

The in vitro concentrations and in vivo doses cannot be directly compared because absorption, metabolism, distribution, and elimination all affect systemic exposure. Since no pharmacokinetic data are available for the UUEE, the actual plasma levels after oral dosing are unknown. So, while both studies showed activity, the exact exposure–response relationship is still unclear.

## 4. Materials and Methods

### 4.1. Plant Material and Extract Preparation

Fresh leaves of *U. urens* were harvested from Setif, northeastern Algeria, in March 2024, corresponding to the flowering stage. The collection site was located at the geographical coordinates (35°49′55.3″ N 5°17′11.0″ E), at an altitude of approximately 1100 m above sea level. Botanical identification was confirmed by Dr. Wafa Nouioua (Department of Plant Ecology and Biology, Faculty of Natural and Life Sciences, Ferhat Abbas University Setif 1, Algeria). A voucher specimen (UFS-2024-10) was deposited at the Laboratory of Phytotherapy Applied to Chronic Diseases. The collected leaves were separated from the stems, air dried in the shade at room temperature, and subsequently finely powdered using an electric grinder. The UUEE was prepared by maceration following the method described by Sharma et al. [64]. In brief, 100 g of powdered *U. urens* leaves were macerated in 1 L of ethanol for seven days at room temperature with periodic shaking. The mixture was then filtered, and the solvent was evaporated under reduced pressure at 45 °C using a rotary evaporator (BÜCHI). The resulting crude extract was stored at 4 °C until further use.

### 4.2. Chemicals

The following chemicals were acquired from Sigma Aldrich: Folin–Ciocalteu reagent, 1,1-diphenyl-2-picrylhydrazyl (DPPH), Na_2_CO_3_, NaNO_2_, AlCl_3_, Complete Freund’s Adjuvant, ethylene diamine tetra-acetic acid (EDTA), Trisma base, 5, 5-dithiobis-(2-nitrobenzoic acid) (DTNB), hydrogen peroxide (H_2_O_2_), reduced glutathione (GSH), trichloroacetic acid (TCA), thiobarbituric acid (TBA), and all other products.

### 4.3. Animals

To perform this study, thirty-five male Wistar rats (200–250 g) were obtained from the Animal House of the Pasteur Institute in Algiers, Algeria. The animals were housed in plexiglass cages under standard laboratory conditions, including a 12-h light/dark cycle, a controlled temperature of 25 °C, and free access to food and water.

### 4.4. LC–HRMS/MS Analysis

LC–HRMS and LC–HRMS/MS analyses were carried out according to Kaouane et al. [65] using an LTQ-Orbitrap Exploris120 mass spectrometer (Thermo Fisher Scientific Spa, Rodano, Italy) equipped with an Vanquish HPLC (Thermo Fisher Scientific Spa, Rodano, Italy). Chromatographic separation was performed at room temperature on a Hypersil GOLD C18 column (150 × 3.0 mm, 3 µm; Thermo Fisher Scientific, Waltham, MA, USA). The mobile phases consisted of water containing 0.1% formic acid (A) and acetonitrile containing 0.1% formic acid (B). The flow rate was 0.450 mL/min, the injection volume was 5 µL, and the autosampler was maintained at 10 °C.

The gradient elution program was as follows: 5% B from 0 to 2 min, 5–50% B from 2 to 10 min, 50% B from 10 to 12 min, 50–95% B from 12 to 18 min, 95% B from 18 to 20 min, and 95–5% B from 20 to 20.1 min, followed by re-equilibration at 5% B until 24 min.

The mass spectrometer was operated in negative ionization mode with a spray voltage of 2.3 kV. The sheath gas, auxiliary gas, and sweep gas were set at 50, 10, and 1 arbitrary units, respectively. The ion transfer tube and vaporizer temperatures were maintained at 320 and 350 °C, respectively, and the RF lens was set to 70%. Full-scan mass spectra were acquired in profile mode over the *m*/*z* range 110–1500 at a resolution of 120,000. The AGC target was set to “Standard”, the maximum injection time to “Auto”, and one microscan was acquired.

Data-dependent MS/MS experiments were performed by selecting the four most intense precursor ions per acquisition cycle. The precursor isolation window was set at 2 *m*/*z*, and MS/MS spectra were acquired in centroid mode at a resolution of 45,000 using stepped normalized collision energies of 20, 40, and 80%. The MS/MS scan range was automatically determined by the instrument. The AGC target was set to “Standard”, the maximum injection time to “Auto”, and one microscan was acquired.

The LC–HRMS/MS data were manually inspected on a peak-by-peak basis using Thermo Scientific FreeStyle software (version 1.8). Metabolite annotation was based on accurate mass, proposed molecular formula, isotopic pattern, and MS/MS fragmentation behavior, which were compared with data reported in the scientific literature.

### 4.5. Antioxidant Assays

#### 4.5.1. DPPH Free Radical Scavenging Assay

The DPPH free radical scavenging activity of UUEE was evaluated according to the method described by Blois et al. [66] with minor modifications. Briefly, in a 96-well microplate a 0.1 mM DPPH solution was prepared in methanol. Aliquots of 160 μL of DPPH solution were mixed with 40 μL of UUEE dissolved in methanol at concentrations of 12.5, 25, 50, 100, 200, 400, and 800 μg/mL. The mixtures were incubated in the dark at room temperature for 30 min, after which absorbance was measured at 517 nm using a UV–Vis microplate reader (EnSpire, PerkinElmer, MA, USA). Butylated hydroxytoluene (BHT) and butylated hydroxyanisole (BHA) were used as positive controls at the same concentrations. Radical scavenging activity was calculated as follows:

DPPH scavenging activity (%) = [(Acontrol − Asample)/Acontrol] × 100

where Acontrol and Asample represent the absorbance of the control and sample, respectively. Results were expressed as IC_50_ values (μg/mL).

#### 4.5.2. ABTS Assay

The ABTS radical scavenging activity was determined according to the method of Re et al. [67]. The ABTS radical cation (ABTS•^+^) was generated by reacting 7 mM ABTS with 2.45 mM potassium persulfate and allowing the mixture to stand in the dark at room temperature for 12–16 h. Prior to analysis, the ABTS•^+^ solution was diluted with ethanol to obtain an absorbance of 0.70 ± 0.02 at 734 nm. Subsequently, 160 μL of ABTS•^+^ solution was mixed with 40 μL of UUEE or standard solution prepared in methanol at concentrations ranging from 3.12 to 200 μg/mL. BHA and BHT were used as positive controls. After incubation for 10 min at room temperature, absorbance was measured at 734 nm using a 96-well microplate reader (EnSpire, PerkinElmer, MA, USA). All measurements were performed in triplicate. The percentage inhibition was calculated according to the following equation:

Inhibition (%) = [(Acontrol − Asample)/Acontrol] × 100

where Acontrol is the absorbance of the control and Asample is the absorbance of the tested sample. Results were expressed as IC_50_ values.

#### 4.5.3. Reducing Antioxidant Capacity (CUPRAC) Assay

The assay was conducted per Apak et al. [68] Briefly, 40 μL sample was mixed with 50 μL 10 mM Cu (II) solution, 50 μL 7.5 mM neocuproine alcoholic solution, and 60 μL 1 M ammonium acetate buffer (pH 7.0). After 1 h incubation, absorbance was measured at 450 nm using a 96-well PerkinElmer EnSpire multimode plate reader. Results are expressed as A_0.5_ (μg/mL) and compared to BHA/BHT standards.

### 4.6. Preliminary Phytochemical Analysis

#### 4.6.1. Determination of Total Phenolic Contents (TPC)

Total phenolic content was determined using the Folin–Ciocalteu method [69]. Briefly, 50 μL of UUEE was mixed with 250 μL of diluted Folin–Ciocalteu reagent (2 M, diluted 1:10) followed by the addition of 250 μL of sodium carbonate solution (7.5%, *w*/*v*). The reaction mixture was incubated at room temperature for 90 min, and absorbance was measured at 765 nm using a UV–Visible spectrophotometer (Shimadzu Corporation, Kyoto, Japan). Gallic acid was used for calibration, and the results were expressed as μg gallic acid equivalents (GAE)/mg dry weight.

#### 4.6.2. Determination of Total Flavonoids Content (TFC)

The total flavonoid content of *U. urens* extract was determined using the modified aluminium chloride method described by Kurkina et al. [70]. For the assay, 1 mL of the extracts or rutin solution was mixed with 0.3 mL of sodium nitrite (NaNO_2_) and 4 mL of distilled water. After 5 min, 0.3 mL of aluminium chloride solution was added, and the mixture was left to react for 6 min. Then, 2 mL of 1 M sodium hydroxide solution was added, and the final volume was adjusted to 10 mL with distilled water after 10 min. The absorbance was measured at 510 nm using UV–Visible spectrophotometer (Shimadzu Corporation, Kyoto, Japan). Rutin was used as the reference standard, and the total flavonoid content was expressed as rutin equivalents.

### 4.7. In Vitro Anti-Arthritic Activity

Briefly, equal volumes (500 μL) of 0.2% bovine serum albumin (BSA) prepared in 20 mM Tris-HCl buffer (pH 6.8) and different concentrations of UUEE were mixed. Diclofenac sodium (250 μg/mL) was used as the positive control. The reaction mixtures were incubated at 37 °C for 20 min and subsequently heated at 65 °C for 10 min. After cooling to room temperature, absorbance was measured at 660 nm using UV–Visible spectrophotometer (Shimadzu Corporation, Kyoto, Japan). The percentage inhibition of protein denaturation was calculated as:

Inhibition (%) = [(Abscontrol − Abssample)/Abscontrol] × 100

### 4.8. In Vitro Cytotoxicity Activity

Cell viability was assessed using the Cell Counting Kit-8 (CCK-8; Dojindo Molecular Technologies, Kumamoto, Japan) according to the manufacturer’s protocol.

HeLa (human cervical carcinoma, ATCC CCL-2) and HaCaT (immortalized human keratinocyte, CLS Cell Lines Service, Eppelheim, Germany) cell lines were cultured in complete Dulbecco’s Modified Eagle Medium (DMEM). Cells were seeded into 96-well plates at a density of 3 × 10^3^ cells per well in 100 μL of culture medium and allowed to adhere for 24 h under standard culture conditions (37 °C, 5% CO_2_, humidified atmosphere).

Following the adhesion period, cells were exposed to serial concentrations of UUEE (200, 100, 50, 25, 12.5, 6.25, 3.125, and 1.56 μg/mL) prepared in DMSO, ensuring that the final DMSO concentration did not exceed 0.1% as reported by Kaouane et al. [59]. Untreated cells receiving culture medium supplemented with 0.1% DMSO served as the negative control, whereas wells containing culture medium and CCK-8 reagent without cells were used as blank controls. Positive control was represented by DMSO 5%.

At the end of the 24 h treatment period, 10 μL of CCK-8 solution was added to each well, followed by an additional 2 h incubation at 37 °C. Cell viability was determined by measuring the absorbance at 450 nm using a microplate reader (Bio-Rad, Thermo Fisher, Hercules, CA, USA).

### 4.9. Comet Assay

The genotoxic potential of UUEE was assessed in HeLa cells using the alkaline comet assay according to the protocol originally described by Singh et al. [71], with minor modifications based on the procedure reported by Dentato et al. [72]. HeLa cells were seeded into 60-mm culture dishes at a density of 4 × 10^5^ cells per dish and allowed to adhere for 24 h before treatment. Subsequently, cells were incubated with UUEE at concentrations of 50, 100, or 200 µg/mL for an additional 24 h.

At the end of the exposure period, cells were harvested, mixed with 1% low-melting-point agarose, and immediately layered onto microscope slides previously coated with 1% normal-melting-point agarose. After solidification, the slides were immersed in a cold lysis solution containing 2.5 M NaCl, 100 mM EDTA, 10 mM Tris, and 1% Triton X-100 (pH 10) for 1 h at 4 °C. DNA unwinding was then carried out by incubating the slides in alkaline electrophoresis buffer (300 mM NaOH, 1 mM EDTA; pH > 13) for 20 min, followed by electrophoresis at 25 V and 300 mA for 20 min. After electrophoresis, the slides were neutralized with 0.4 M Tris buffer (pH 7.5) and stained with ethidium bromide (5 µg/mL; Sigma-Aldrich, Darmstadt, Germany).

DNA migration was examined under a fluorescence microscope, and comet images were analyzed using the OpenComet plugin (version 2.9.0) integrated into the ImageJ version 1.38 software package. For each experimental condition, a total of 50 randomly selected, non-overlapping nucleoids (25 from each of two replicate slides) were scored. All experiments were independently repeated at least twice. DNA damage was quantified using three standard comet assay descriptors: percentage of DNA in the tail, tail moment, and Olive tail moment.

### 4.10. RT-qPCR

To investigate the immunomodulatory activity of UUEE, HaCaT keratinocytes were pretreated with the extract prior to zymosan stimulation, and the mRNA expression levels of the pro-inflammatory cytokines IL-1β and IL-6 were quantified by real-time quantitative PCR (RT-qPCR), following the protocol described by Kaouane et al. [65].

HaCaT cells were seeded in 60 mm culture dishes at a density of 2.5 × 10^5^ cells per dish and incubated overnight to allow cell attachment. Cells were then exposed for 24 h to UUEE extract (50 μg/mL), vehicle control (0.1% DMSO), or quercetin (1.5 μM; Q0125, Sigma-Aldrich, Darmstadt, Germany), which served as the positive control.

Following the pretreatment period, cells were stimulated with zymosan (75 μg/mL; Z4250, Sigma-Aldrich, Darmstadt, Germany) for an additional 24 h to induce an inflammatory response. Control groups consisted of untreated cells, cells treated with vehicle (0.1% DMSO), and cells pretreated with UUEE followed by vehicle instead of zymosan, allowing evaluation of the extract in the absence of inflammatory stimulation.

Total RNA was isolated using the VWR Total RNA Purification Kit according to the manufacturer’s protocol. RNA quantity and purity were assessed by spectrophotometric analysis. For cDNA synthesis, 1 μg of total RNA was reverse-transcribed using the Vazyme HiScript Reverse Transcription Master Mix (Vazyme, Nanjing, China), following the manufacturer’s recommendations.

Gene expression analysis was performed by RT-qPCR using the Vazyme 2× Taq Pro Universal SYBR qPCR Master Mix (Vazyme, Nanjing, China) on an Applied Biosystems StepOnePlus™ Real-Time PCR System (Applied Biosystems, Darmstadt, Germany). Human IL-1β primers (forward: 5′-CCACAGACCTTCCAGGAGAATG-3′; reverse: 5′-GTGCAGTTCAGTGATCGTACAGG-3′), human IL-6 primers (forward: 5′- AGACAGCCACTCACCTCTTCAG-3′; reverse: 5′-TTCTGCCAGTGCCTCTTTGCTG-3′) and GAPDH primers (forward: 5′-TTGCCATCAATGACCCCTTCA-3′; reverse: 5′-CGCCCCACTTGATTTTGGA-3′), used as the reference gene, were obtained from Euroclone. Amplification was carried out with an initial denaturation step at 95 °C for 30 s, followed by 40 cycles consisting of 95 °C for 5 s and 60 °C for 30 s. Relative gene expression levels of IL-1β and IL-6 were calculated using the 2−ΔΔCt method after normalization against GAPDH. All reactions were performed in technical triplicate.

### 4.11. In Vivo Anti-Arthritic Activity

#### 4.11.1. CFA Induced Arthritis in Rats

Arthritis was induced according to Parmar et al. [73], with slight modifications. Briefly,100 μL of CFA (containing 10 mg/mL of heat-killed *Mycobacterium tuberculosis*) was injected subcutaneously into left hind paw of all the animals on day 0.

The animals were divided into five groups consisting of seven animals per group: (*n* = 7).

Group 1: Designated as normal control, healthy rats received distilled water for 21 days without RA induction.

Group 2: [CFA group (negative control)]: Rheumatoid arthritis rats were given distilled water by oral gavage for 21 days.

Group 3: The indomethacin group was treated with oral indomethacin (5 mg/kg/day, daily p.o.) for 21 days.

Group 4: Low-dose—rats were treated with UUEE (150 mg/kg/day p.o.).

Group 5: High-dose—rats were treated with UUEE (300 mg/kg/day p.o.).

#### 4.11.2. Drug Administration

Treatments were administered daily for 21 consecutive days of arthritis induction. UUEE and indomethacin were dissolved in saline containing 0.1% tween 80 just before oral administration. Measurement of arthritis score, body weight and paw volume were performed every two days until day 21. At the end, the rats were euthanized, and blood samples were collected via retro-orbital puncture for the analysis of various biochemical and hematological parameters.

#### 4.11.3. Measurement of Paw Swelling, Arthritis Score and Weight Changes

The Vernier caliper was used to measure the paw and paw thickness. The clinical severity of arthritis was assessed using a visual scoring system based on observable signs and symptoms on a scale from 0 to 4 based on observable signs and symptoms: 0 = no change; 1 = mild erythema or swelling of the digits; 2 = moderate swelling and erythema; 3 = severe swelling and erythema involving the ankle; and 4 = ankylosis and inability to bend the ankle [4]. Weight changes were recorded for each group every two days, from day 0 to 21.

#### 4.11.4. Evaluation of Oxidation Status

At the end of the treatment period, the rats were sacrificed by cervical dislocation, and their livers, spleens were excised and rinsed. tissues homogenates were prepared using 0.15 M KCl buffer at −4 °C, the tissue supernatant was used to estimate the levels of oxidative stress markers, including reduced glutathione (GSH), malondialdehyde (MDA), catalase (CAT), and nitric oxide (NO).

The reduced glutathione (GSH) of both tissue homogenates was determined by the method of Beutler et al. [74]. GSH content was quantified by its reduction of 5,5′-dithio-bis (2-nitrobenzoic acid) (DTNB) to the yellow TNB anion (ε = 14,150 M^−1^ cm^−1^), measured at 412 nm. Briefly, 25 μL tissue homogenate was added to 5 mL phosphate buffer (0.1 M, pH 8). Then, 1.4 mL of this mixture was combined with 10 μL 0.01 M DTNB, vortexed, and incubated for 5 min. Absorbance was read at 412 nm, and GSH levels calculated using the molar extinction coefficient.

Hepatic and splenic MDA levels were quantified via TBARS formation upon reaction with thiobarbituric acid (TBA), measured spectrophotometrically [75]. Briefly, 0.5 mL sample was mixed with 0.5 mL 20% trichloroacetic acid (TCA) and 1 mL 0.67% TBA, then heated in a boiling water bath for 15 min. After cooling, 4 mL n-butanol was added, vortexed, and centrifuged (3000 rpm, 15 min). The organic phase absorbance was read at 530 nm, with MDA equivalents calculated using ε = 1.56 × 10^5^ M^−1^ cm^−1^.

Catalase enzymatic activity was estimated according to the method of Aebi [76], which monitors the breakdown of H_2_O_2_ to give water and oxygen molecular in the presence of enzyme source (homogenate). Briefly, 983.5 μL of 0.091 M H_2_O_2_ in potassium phosphate buffer (0.1 M, pH 7.2) was added to 16.5 μL homogenate or plasma. The reaction rate was determined from the decrease in absorbance at 240 nm. Enzymatic activity was determined by using the molar extinction coefficient 43.6 M^−1^ cm^−1^.

The measurement of nitrite ion levels provides an index of nitric oxide radical production. According to the adopted protocol of Abdelouhab et al. [77]. NO was estimated by adding to 100 μL of tissue homogenate an equal volume (50 μL) of 1% sulfanilamide dissolved in 5% phosphoric acid and 0.1% NEDD solution. The mixture was incubated at room temperature for 25 min in the dark, and the absorbance was read at 543 nm. A standard curve of nitrite was prepared under the same conditions. The unknown concentration of NO in the tissue homogenates was then calculated from the linear regression equation (Y= 0.0051x + 0.0361, R^2^ = 0.997), and the results are expressed as μM nitrite equivalent in spleen/liver homogenate.

#### 4.11.5. Determination of Hematological and Biochemical Parameters

Blood samples collected in EDTA tubes were analyzed for complete blood count (CBC) using an automated hematology analyzer. For serum separation, blood samples were centrifuged at 3000× *g* for 15 min. The serum was subsequently used to assess liver and renal function biomarkers.

#### 4.11.6. Histological Changes

After serum collection, the rats were sacrificed under deep anesthesia. The paws were then excised for histological analysis, fixed in 10% buffered formalin, and decalcified in nitric acid. All samples were then transported to the Pathology Laboratory of the CAC–Sétif, Algeria, for further processing and analysis. Additionally, histological examinations of the liver were conducted to assess potential toxic effects. Histological examination was performed according to the standard experimental protocol of the laboratory, as described by Bancroft and Gamble [78].

### 4.12. Statistical Analysis

Statistical analyses were performed using GraphPad Prism software (version 8.0, GraphPad Software, San Diego, CA, USA). Data are presented as mean ± standard deviation (SD). Differences between two groups were evaluated using the unpaired Student’s *t*-test. Behavioural parameters were analyzed using two-way analysis of variance (ANOVA) followed by Tukey’s multiple comparison test. Oxidative stress biomarkers were analyzed using one-way ANOVA followed by Dunnett’s post hoc test. Cytotoxicity, comet assay, gene expression, antioxidant activity, and protein denaturation data were analyzed using one-way ANOVA followed by Tukey’s multiple comparison test. Differences were considered statistically significant at *p* < 0.05.

## 5. Conclusions

In conclusion, the results of the present study demonstrate that UUEE possesses significant antioxidant, anti-inflammatory, and anti-arthritic properties. The LC–MS phytochemical characterization revealed the presence of several bioactive compounds, including phenolic compounds and flavonoids, which may contribute to the biological activities observed. The extract effectively inhibited protein denaturation in vitro, showed a favorable cytotoxicity profile in HaCaT cells, and reduced zymosan-induced IL-1β and IL-6 expression, highlighting its potential to modulate inflammatory responses. Furthermore, UUEE reduced paw swelling, arthritic scores, and joint damage in Freund’s Complete Adjuvant-induced arthritic rats, while also preventing cachexia and improving hematological, biochemical, and antioxidant parameters. These beneficial effects appear to be closely linked to the presence of polyphenols and flavonoids identified through LC-MS analysis, which contribute to membrane stabilization, modulation of inflammatory mediators, and attenuation of oxidative stress. Overall, UUEE may represent a promising natural candidate for the prevention or adjunctive management of rheumatoid arthritis, warranting further preclinical and clinical investigation.

## Figures and Tables

**Table 1:** Identification of metabolites detected in the UUEE by LC-ESI-HRMS in negative ionization mode.

No.	Name	R*_t_*	Delta ppm	Molecular Formula	[M-H]^−^	MS/MS
1	quinic acid	5.37	4.48	C_7_H_12_O_6_	191.0560	173.0456, 127.0402, 111.0453, 93.0347, 85.0296
2	esculetin	5.43	4.76	C_9_H_6_O_4_	177.0190	149.0246, 133.0297, 120.9624, 105.0347, 89.0399
3	5-*O*-caffeoylquinic acid	5.65	2.21	C_16_H_18_O_9_	353.0880	191.0560, 179.0349, 173.0456
4	phenylethyl primeveroside	5.96	2.49	C_19_H_28_O_10_	[M + FA]- 461.1660	415.1607, 161.0456, 131.0350, 113.0245, 101.0245
5	*p*-coumaroylquinic acid	6.29	3.28	C_16_H_18_O_8_	337.0920	191.0561, 173.0455, 163.0402, 119.0505, 93.0347
6	N-acetylphenylalanine	6.34	4.56	C_11_H_13_NO_3_	206.0820	164.0717, 147.0453, 116.8792, 103.1589, 91.0554
7	putative terpenoid *O*-hexoside	6.54	0.79	C_25_H_40_O_12_	531.24408	369.1112, 223.1338, 205.3187
8	putative terpenoid *O*-hexoside isomer	6.78	0.79	C_25_H_40_O_12_	531.24408	223.1343, 205.3187
9	azelaic acid	7.45	4.92	C_9_H_16_O_4_	187.0980	169.0870, 143.1079, 125.0973, 97.0659
10	astragalin	7.49	1.99	C_21_H_20_O_11_	447.0940	285.0402, 255.0300, 227.0349, 151.0038
11	diosmetin-7-*O*-rutinoside	7.57	1.75	C_28_H_32_O_15_	607.1670	299.0561, 284.0326, 107.5117
12	putative oxygenated terpenoid glycoside	7.61	1.01	C_25_H_40_O_11_	515.24921	353.6278, 148.9725, 131.0351, 113.0245, 101.0244, 89.0244
13	geranylphenylacetate glycoside	7.76	1.29	C_25_H_38_O_11_	513.23370	333.5540, 189.1286, 164.8556, 119.0350, 101.0244, 89.0244
14	geranylphenylacetate glycoside isomer	7.88	1.29	C_25_H_38_O11	513.23370	333.5540, 189.1286, 164.8556, 119.0350, 101.0244, 89.0244
15	oxo-dodecanedioic acid	8.00	4.36	C_12_H_20_O_5_	243.1240	199.1339, 181.1233, 155.1443, 127.7469, 99.0088
16	phytofuran isomer	8.28	3.57	C_18_H_32_O_6_	343.2130	325.2024, 209.1183
17	sebacic acid	8.41	4.50	C_10_H_18_O_4_	201.1130	201.1132, 183.1026, 139.1130
18	indole-3-pyruvic acid	8.71	4.26	C_11_H_9_NO_3_	202.0510	158.12688, 130.06622, 102.50820
19	trihydroxy-octadecadienoic acid	9.21	2.55	C_18_H_32_O_5_	327.2180	327.2180, 291.1968, 211.1339, 171.1027, 137.0973
20	undecanedioic acid	9.28	4.62	C_11_H_20_O_4_	215.1290	197.1183, 153.1286
21	trihydroxy-octadecenoic acid	9.67	2.18	C_18_H_34_O_5_	329.23297	311.2230, 229.1446, 211.1339, 171.1027, 139.1130
22	dodecanedioic acid	10.08	4.30	C_12_H_22_O_4_	229.1440	211.13387, 185.15446, 167.14410, 138.96494
23	linolenic acid hydroperoxide	10.45	2.44	C_18_H_30_O_4_	309.20679	291.1967, 273.18658, 223.13420, 211.1343, 197.1184, 171.10265, 127.11320
24	phytoprostane derivative	10.75	2.52	C_18_H_28_O_4_	307.19116	289.18146, 263.00531, 245.19112, 185.1182
25	linolenic acid hydroperoxide isomer I	11.19	2.44	C_18_H_30_O_4_	309.20679	291.1967, 273.18658, 223.13420, 211.1343, 197.1184, 171.10265, 127.11320
26	linolenic acid hydroperoxide isomer II	11.40	3.22	C_18_H_30_O_4_	309.20691	291.1967, 273.18658, 223.13420, 211.1343, 197.1184, 171.10265, 127.11320
27	dihydroxy-octadecenoic acid	12.06	3.59	C_18_H_34_O_4_	313.2390	295.22803, 277.21741, 183.1390, 171.10262, 155.10786, 127.11289
28	putative ascorbyl fatty acid ester	12.24	0.95	C_22_H_38_O_7_	413.25433	255.15956, 201.74132, 150.90414, 116.07177
29	oxo-octadecadienoic acid	13.52	2.69	C_18_H_30_O_3_	293.21204	275.2018, 223.1704, 231.21198, 185.11859, 183.13910, 171.10271, 121.10240
30	oxo-octadecadienoic acid isomer I	13.70	2.69	C_18_H_30_O_3_	293.21191	293.2123, 275.2017, 235.1700, 231.2120, 211.1338, 183.1390, 171.1027, 120.7341
31	putative oxygenated triterpenoid/limonoid derivative	13.76	−1.85	C_34_H_44_O_9_	595.28906	415.2257, 315.0488, 279.2329
32	brevifolincarboxylic acid	14.18	2.81	C_13_H_8_O_8_	291.19629	247.2066, 229.1960, 219.1391, 191.1443, 163.1130, 147.0822
33	hydroxy-octadecadienoic acid	14.67	2.70	C_18_H_32_O_3_	295.2280	295.2280, 277.2174, 211.4799, 195.1388, 171.1026
34	oxo-octadecadienoic acid isomer II	15.04	3.24	C_18_H_30_O_3_	293.21207	293.2123, 275.2017, 235.1700, 231.2120, 211.1338, 183.1390, 171.1027, 120.7341
35	ricinoleic acid	15.18	3.33	C_18_H_34_O_3_	297.2430	296.9946, 182.9548
36	hydroxy-octadecadienoic acid isomer	15.79	3.01	C_18_H_32_O_3_	295.22766	295.2280, 277.2172, 211.4048
37	unknown	15.83	−3.52	C_29_H_38_O_6_	481.25677	253.21735, 245.04326, 171.00641, 152.99593
38	putative oxygenated steroid	16.00	2.87	C_21_H_28_O_2_	311.2014566	293.19049, 267.33282, 149.0972, 119.71858
39	linolenic acid	16.98	3.44	C_18_H_30_O_2_	277.21716	259.20663, 233.22751, 159.31667, 132.99203
40	linoleic acid	17.69	3.13	C_18_H_32_O_2_	279.23273	261.22226, 216.71979, 122.83182

**Table 2:** Antioxidant activity of UUEE determined by DPPH, ABTS radical scavenging and CUPRAC assays compared with standards. Values represent mean ± SD (*n* = 3). Statistical analysis was performed using one-way ANOVA (**** *p* < 0.0001).

Compound	DPPH IC_50_ ± SD (µg/mL)	ABTS IC_50_ ± SD (µg/mL)	CUPRAC Assay A_0.5_ ± SD (µg/mL)
UUEE	445.10 ± 7.27 ****	212.49 ± 8.91 ****	166.46 ± 6.49 ****
BHA	6.14 ± 0.41	1.29 ± 0.30	5.35 ± 0.71
BHT	12.99 ± 0.41	1.81 ± 0.10	8.97 ± 3.94

**Table 3:** Total phenolic and flavonoid content of UUEE.

Extract	Total Phenolics (mg GAE/g DW)	Total Flavonoids (mg RE/g DW)
UUEE	16.86 ± 3.92	2.46 ± 0.01

**Table 4:** Hematological parameters in CFA-induced arthritic rats treated with UUEE. The values are expressed as mean ± SEM (*n* = 7). * *p* < 0.05, ** *p* < 0.01, *** *p* < 0.001, **** *p* < 0.0001 vs. the CFA group. WBC: white blood cells; RBC: red blood cells; HGB: hemoglobin; HCT: hematocrit; PLT: platelets; ASAT: aspartate aminotransferase; ALAT: alanine aminotransferase; TRI: triglycerides.

Parameters	CFA Group	Control Group	Indomethacin	UUEE (300 mg)	UUEE (150 mg)
Hematological values					
WBC (10^9^/L)	15.46 ± 0.93	13.68 ± 1.27	13.53 ± 0.43	13.86 ± 0.67	14.6 ± 1.02
RBC (10^12^/L)	7.55 ± 0.25	8.58 ± 0.22	7.84 ± 0.07	8.44 ± 0.07	7.60 ± 0.35
HGB (g/dL)	14.66 ± 0.39	15.93 ± 0.22	15.35 ± 0.41	15.11 ± 0.21	14.8 ± 0.37
HCT (%)	42.73 ± 1.02	47.46 ± 0.65	45.06 ± 1.93	45.38 ± 0.85	43.9 ± 2.44
PLT (10^9^/L)	993 ± 19.65	747 ± 12.62 ****	783.33 ± 16.66 ****	844 ± 14.43 ****	907 ± 10.71 ****
Biochemical values					
Urea (g/L)	0.38 ± 0.04	0.32 ± 0.01	0.3 ± 0.03	0.28 ± 0.02	0.33 ± 0.01
Creatinine (mg/dL)	8.42 ± 0.24	4.37 ± 1.4	3.46 ± 0.49	3.2 ± 0.13	5.1 ± 1.27
TRI (g/L)	1.03 ± 0.10	1.04 ± 0.22	1.3 ± 0.13	1.34 ± 0.14	0.81 ± 0.02
ASAT (u/L)	87.41 ± 1.21	65.01 ± 5.28 ***	83.25 ± 3.26	79.73 ± 3.09 ****	87.96 ± 8.44
ALAT (u/L)	58.23 ± 1.09	44.99 ± 1.6	44.1 ± 1.18	40.07 ± 2.35 **	44.18 ± 3.42 *

## Data Availability

The data presented in this study are available on request from the corresponding author.
